# The Role of Long Non-Coding RNAs in Breast Cancer Diagnosis and Treatment Based on Evidence from Clinical Studies: A Scoping Review

**DOI:** 10.3390/epigenomes10040065

**Published:** 2026-10-07

**Authors:** Menelaos Zafrakas, Panayiota Papasozomenou, Ioannis Gavalas, Christos Emmanouilidis, Maria Chatzidimitriou

**Affiliations:** 1 School of Health Science, International Hellenic University, 57400 Thessaloniki, Greece; papasozomenou248@gmail.com (P.P.); chdimitr@ihu.gr (M.C.); 2 Department of Medical Oncology, European Interbalkan Medical Center, 55535 Thessaloniki, Greece; igava@auth.gr (I.G.); chrem@interbalkan-hosp.gr (C.E.)

**Keywords:** long non-coding RNA, lncRNA, breast cancer, scoping review

## Abstract

**Background/Objectives:** The aim of this scoping review was to investigate the current role and possible clinical applications of long non-coding RNAs (lncRNAs) in breast cancer diagnosis and treatment. **Methods:** A thorough search of the PubMed/MEDLINE and Scopus/EMBASE databases was carried out in accordance with the PRISMA-ScR guidelines. The inclusion criteria were as follows: Studies with lncRNA analyses of specimens from breast cancer patients and clinically relevant studies. The exclusion criteria were as follows: in silico and in vitro studies involving animal models; reviews; case series; case reports; comments; editorials; and papers not written in English. The study protocol was registered in the Open Science Framework. **Results:** After the identification of 1139 records, 312 publications were retrieved and 228 articles were selected for data extraction. Data analysis and the synthesis of evidence showed that lncRNA studies were conducted in breast tumor specimens in 212 studies and in blood samples from breast cancer patients in 20 studies. The most commonly used method was quantitative RT-PCR (in 216 studies), while many studies were complemented by in vitro (*n* = 177) and animal experiments (*n* = 75). There were 39 studies that included at least 100 specimens from breast cancer patients. **Conclusions:** At present, lncRNA analysis is not used in routine clinical practice. Clinically relevant findings coupled by results from in vitro and animal experiments suggest that various lncRNAs after appropriate validation may be used as biomarkers for breast cancer diagnosis and treatment, most notably HSAT164, CCAT2, HOTAIR, and MALAT1. To this end, larger studies involving clinical specimens from clinically relevant subgroups of breast cancer patients will be needed.

## 1. Introduction

Breast cancer is the most commonly diagnosed malignancy and the leading cause of cancer death in women worldwide, followed by lung and colorectal cancer for both cancer incidence and mortality [1]. Since the early 1990s, breast cancer mortality rates have decreased in many high-income countries as a consequence of the widespread use of mammographic screening leading to early diagnosis and due to increasingly effective treatment methods [1,2]. In turn, decreased mortality rates have led to an increased prevalence of long-term breast cancer survivors, while recurrences continue to occur even more than three decades after primary diagnosis [3]. Circulating tumor cells (CTCs) and tumor cells in the bone marrow of breast cancer patients have been detected years before disease recurrence [3,4,5,6], suggesting that disease persists outside of the breast or local lymph nodes in a clinically latent state before it develops into manifest metastases, a concept also referred to as tumor dormancy [5]. Hence, it is not surprising that breast cancer remains the most common cause of death after breast cancer diagnosis, though other entities, mainly heart and cerebrovascular disease, represent a significant cause of mortality among breast cancer survivors [7,8].

For many years in the past, the decision of whether to administer systemic cytotoxic chemotherapy or not to patients with hormone receptor-positive early breast cancer has been a challenge in everyday clinical practice, as clinicians had to balance between the possibility of over-treatment with associated toxicity and under-treatment with the risk of early disease recurrence [9,10]. The development of multi-gene expression assays at the mRNA level has been a paradigm shift in the treatment of early breast cancer, as these assays now have paramount importance in the decision of whether to administer chemotherapy or not to patients with early luminal breast cancer [11,12]. Other than mRNA, in recent years, research has focused on investigating the potential role of long non-coding RNAs (lncRNAs) in breast cancer pathogenesis and tumor progression, with possible applications in diagnosis and treatment. Several lines of evidence have implicated lncRNAs in the regulation of cancer stem cells and the tumor microenvironment [13,14], the cell cycle [15], inflammation in breast cancer [16], and the possibility to be used as therapeutic targets [17,18]. However, it is unclear to clinicians if lncRNAs already have clinical applications in breast cancer diagnosis and treatment. Therefore, the objective of this scoping review was to investigate the current role and possible clinical applications of lncRNA analyses in breast cancer diagnosis and treatment.

## 2. Results

### 2.1. Search Results

**Figure 1:**
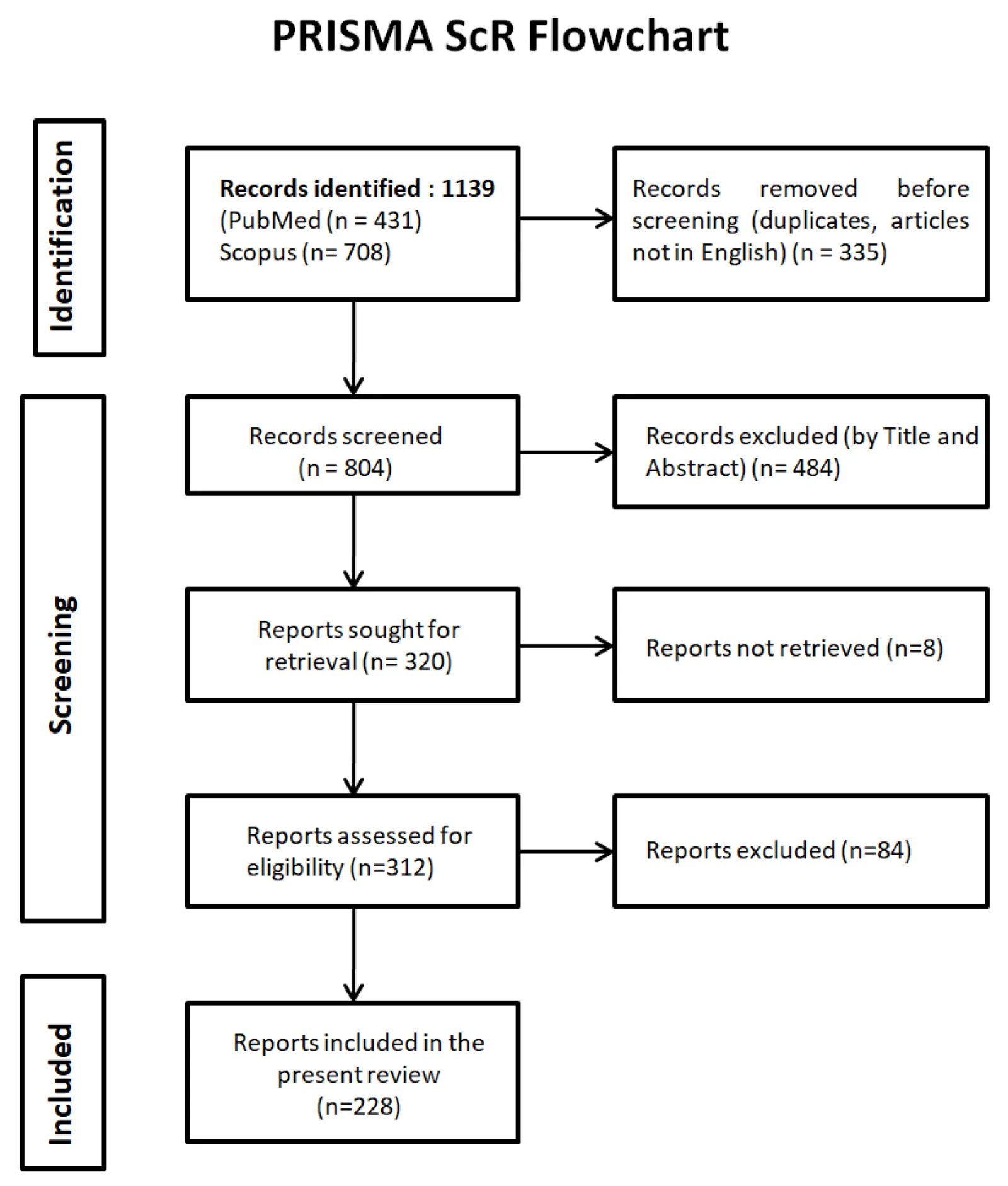
Preferred Reporting Items for Systematic Reviews and Meta-analyses extension for Scoping Reviews (PRISMA-ScR) flowchart.

Figure 1 presents a flow chart of the study selection process and search results, in accordance with the Preferred Reporting Items for Systematic Reviews and Meta-analyses extension for Scoping Reviews (PRISMA-ScR) [19,20]. In detail, 1139 entries (431 in PubMed and 708 in Scopus) were identified by using the search terms “non-coding RNA” AND “breast cancer”, AND “ncRNA” AND “breast cancer”, with the limits “title” for PubMed and “Article title” for Scopus. First, duplicates, overlapping publications, and articles not written in English (*n* = 335) were removed. Next, the titles and/or abstracts of the remaining 804 records were evaluated, leading to the exclusion of 484 studies according to the exclusion criteria and 320 studies to be sought for retrieval. In total, 312 studies were retrieved and, following evaluation of their full-text, 84 studies were excluded based on the eligibility criteria. Finally, 228 articles were included [21,22,23,24,25,26,27,28,29,30,31,32,33,34,35,36,37,38,39,40,41,42,43,44,45,46,47,48,49,50,51,52,53,54,55,56,57,58,59,60,61,62,63,64,65,66,67,68,69,70,71,72,73,74,75,76,77,78,79,80,81,82,83,84,85,86,87,88,89,90,91,92,93,94,95,96,97,98,99,100,101,102,103,104,105,106,107,108,109,110,111,112,113,114,115,116,117,118,119,120,121,122,123,124,125,126,127,128,129,130,131,132,133,134,135,136,137,138,139,140,141,142,143,144,145,146,147,148,149,150,151,152,153,154,155,156,157,158,159,160,161,162,163,164,165,166,167,168,169,170,171,172,173,174,175,176,177,178,179,180,181,182,183,184,185,186,187,188,189,190,191,192,193,194,195,196,197,198,199,200,201,202,203,204,205,206,207,208,209,210,211,212,213,214,215,216,217,218,219,220,221,222,223,224,225,226,227,228,229,230,231,232,233,234,235,236,237,238,239,240,241,242,243,244,245,246,247,248], and extraction of the data followed.

### 2.2. Year of Publication and Geographical Area of Included Studies

Regarding the year of publication, all studies except one were published during the last 12 years, with the number of studies reaching a maximum in 2019 and decreasing thereafter. In detail, one study was published in 2009 [242], three studies in 2013 [239,240,241], three studies in 2014 [236,237,238], eight studies in 2015 [228,229,230,231,232,233,234,235], 12 studies in 2016 [216,217,218,219,220,221,222,223,224,225,226,227], 24 studies in 2017 [192,193,194,195,196,197,198,199,200,201,202,203,204,205,206,207,208,209,210,211,212,213,214,215], 25 studies in 2018 [166,167,168,169,170,171,172,173,174,175,177,178,179,180,181,182,183,184,185,186,187,188,189,190,191], 40 studies in 2019 [125,126,127,128,129,131,132,133,134,135,136,137,138,139,140,141,142,143,144,145,146,147,148,149,150,151,152,153,154,155,156,157,158,159,160,161,162,163,164,165], 37 studies in 2020 [74,90,91,92,93,94,95,96,97,98,99,100,101,102,103,104,105,106,107,108,109,110,111,112,113,114,115,116,117,118,119,120,121,122,123,124,130], 34 studies in 2021 [54,55,56,57,58,59,60,61,62,63,64,65,66,67,68,69,70,71,72,73,75,76,77,78,80,81,82,83,84,85,86,87,88,89], 13 studies in 2022 [42,43,44,45,46,47,48,49,50,51,52,53,79], 11 studies in 2023 [25,32,33,34,35,36,37,38,39,40,41], eight studies in 2024 [22,24,26,27,28,29,30,31], six studies in 2025 [21,23,176,243,244,245], and three studies until April 2026 [246,247,248]. A timeline chart showing the number of publications over time is presented in Supplementary Figure S1.

Regarding the country of first author’s affiliation, 177 out of 228 studies (77.6%) came from China and the remaining 51 from the rest of the world, as follows: (a) 204 studies came from Asia, i.e., 177 from China [23,25,26,27,29,30,34,35,38,39,41,42,43,44,45,48,49,50,51,52,53,55,56,57,58,59,61,63,66,67,68,69,70,73,74,75,77,78,79,82,83,84,85,86,87,88,89,91,92,93,95,96,98,99,102,103,104,105,106,107,108,109,110,111,113,114,115,116,117,118,119,121,122,123,124,127,128,129,130,131,132,133,134,135,137,138,139,140,141,142,144,147,148,149,150,152,153,154,155,156,157,158,159,160,161,162,163,164,165,166,167,169,170,171,172,173,174,175,176,177,178,179,180,181,182,183,184,185,186,187,188,189,191,192,193,194,195,196,197,198,199,200,201,202,203,204,206,207,209,210,211,212,213,214,215,216,217,218,219,220,221,223,224,225,226,227,228,229,230,231,232,233,235,236,238,244], 19 from Iran [21,22,28,33,40,46,56,80,81,100,101,112,120,136,145,146,205,240,246], two from Saudia Arabia [32,60], one from India [37], one from Lebanon [97], one from South Korea [168], and one from Taiwan [148]; (b) 11 studies came from Europe, i.e., three from Denmark [62,234,239], one from Spain [24], one from Greece [36], one from Russia [47], one from Romania [71], one from Italy [94], one from France [208], one from Germany [237], and one from the Netherlands and Sweden [243]; (c) six studies came from North and South America, i.e., two from the USA [222,241], two from Brazil [72,126], one from Canada [242], and one from Mexico [125]; (d) eight studies came from Africa, all from Egypt [31,64,76,90,190,245,247,248]; and (e) one study came from Oceania, i.e., from New Zealand [143]. The geographical distribution of studies is diagrammatically presented in Supplementary Figure S2.

### 2.3. Clinical Relevance and Characteristics of Included Studies

Regarding the type of tissue specimen analyzed, lncRNA analysis was carried out in breast tumor specimens in 212 studies (Table 1 and Supplementary Table S1) and in blood samples from breast cancer patients in 20 studies (Table 2), i.e., 10 studies involving serum, seven studies involving plasma, and three studies involving whole blood. In four studies, both blood and breast tumor specimens were analyzed. Regarding the disease stage, in most studies, specimens from patients with primary invasive breast cancer (stages I–III) were analyzed, while specimens from patients with DCIS (stage 0) and metastatic breast cancer (stage IV) were included only rarely. The most commonly used method (in 216 studies) was quantitative reverse transcription polymerase chain reaction (qRT-PCR). Many studies were complemented by in vitro (*n* = 177 or 77.6% of included studies) and animal experiments (*n* = 75 or 32.9% of included studies) (Supplementary Table S2).

#### 2.3.1. Studies with Non-Coding RNA Analyses in Breast Cancer Tissue

Long non-coding RNA analyses were carried out in breast tumor tissue with or without normal breast tissue specimens in 212 studies. The number of specimens analyzed ranged between 18 and 99 in 179 studies; there were 23 studies with 100–200 specimens [38,48,58,61,62,78,88,93,125,146,149,154,161,162,170,199,201,207,218,224,232,234,239] and only 10 studies [84,124,169,195,200,216,217,223,227,241] with more than 200 specimens analyzed.

In Table 1, the 33 largest studies [38,48,58,61,62,78,84,88,93,124,125,146,149,154,161,162,169,170,195,199,200,201,207,216,217,218,223,224,227,232,234,239,241] of lncRNA analyses with at least 100 breast tumor tissue specimens with or without normal breast tissue samples are presented in descending order of the number of specimens. The following lncRNAs were identified as potential diagnostic biomarkers and/or therapeutic targets in breast cancer tissue: HSAT164 [84], CCAT2 [241], C19orf33, C3orf52 C15orf48, and C4orf19 [195]; MVIH [217], EPB41L4A-AS2 [223], MALAT1 [216,218,247], HUMT [124], Linc-ITGB1 [200], BANCR [169], MEG3 [227], CRALA [201], MIAT [170], HOTAIR [239], RHPN1-AS1 [161], LncKLHDC7B [125], LUCAT1 [162], MIR-1 [232], RACGAP1P [88], MCF2L-AS1 [48], COX10-DT [38], HOXA-AS2 [93], Lnc-BTG3-7:1 [61], ST8SIA6-AS1, lnc-HIST1H2BJ-5:1, lnc PRICKLE2-3:2, RP1-86C11.7, RP11-15F12.1, ZNF670-ZNF695, and lnc-STRN3-12:1 [58]; and LINC01287 [149], H19 [146], NORAD [78], AFAP1-AS1 [224], SPRY4-IT1 [154], TUG1 [199], and HOXA11-AS [207]. Supplementary Table S1 presents the 179 studies with fewer than 100 breast tumor tissue specimens with or without normal breast tissue samples, in descending order of the number of specimens.

**Table 1:** The largest studies with non-coding RNA analyses in breast tumor specimens and/or normal breast tissue (in descending order of the number of specimens).

Study	BC Stage	Number of Specimens	Primary Method	Main Findings and Conclusions
Xu et al. 2021 [84]	P	5634 BC and 6308 controls	lncRNA array	LncHSAT164 is highly expressed in BC and is a potential therapeutic target in BC.
Redis et al. 2013 [241]	P	30 BC, 18 BC and 997 BC	qRT-PCR	LncRNA’s CCAT2 expressioncan predict clinical outcomes for a specific subgroup of BC patients, for which its high levels can indicate that these patients will not benefit from CMF adjuvant chemotherapy.
Lang et al. 2017 [195]	P	286 BC	qRT-PCR	The expression of C19orf33, C3orf52 and C15orf48 was significantly upregulated and C4orf19 was significantly downregulated in multicentric BC. These ncRNAs may be used as biomarkers for multicentric BC.
Lei et al. 2016 [217]	P	250 BC and 50 normal	qRT-PCR	LncRNA MVIH is expressed at high levels in BC and is related to poor prognosis.
Xu et al. 2016 [223]	P	250 BC and 50 normal	qRT-PCR	LncRNA EPB41L4A-AS2 expression is associated with favorable disease outcomes.
Huang et al. 2016 [216]	P	237 BC and 33 normal	qRT-PCR	LncRNA MALAT1 is upregulated in BCandis associated with ER+/PR+; its overexpression is associated with poorer RFS in tamoxifen-treated patients.
Zheng et al. 2020 [124]	P	228 BC	qRT-PCR	LncRNA HUMT was identified as a novel TNBC lymph node metastasis-associated lncRNA and a possible therapeutic target in TNBC.
Li et al. 2017 [200]	P	224 BC and 224 normal	qRT-PCR	LncRNA linc-ITGB1 is underexpressed in BC; its expression is associated with lymph node metastasis, pathological differentiation, TNM stage and poorer disease-free and overall survival.
Jiang et al. 2018 [169]	P	216 BC and 216 normal	qRT-PCR	LncRNA BANCR is overexpressed in BC, and its overexpression is associated with poor overall and recurrence-free survival rates.
Zhang et al. 2016 [227]	P	207 BC and 207 normal	qRT-PCR	LncRNA MEG3 may be an independent prognostic indicator for BC patients.
Li et al. 2017 [201]	P and M	179 BC	qRT-PCR	LncRNA CRALA is associated with poor clinical response to chemotherapy and may be considered an independent prognostic factor for PFS in BC patients.
Li et al. 2018 [170]	P	170 BC	qRT-PCR	LncRNA MIAT could serve as a potential biomarker and promising therapeutic target for ER-positive breast cancer.
Sørensen et al. 2015 [234]	P	164 BC	Microarray analysis	LncRNA profiles provide additional prognostic information and may contribute to the identification of early BC patients eligible for adjuvant therapy.
Sørensen et al. 2013 [239]	P	164 BC	Microarray analysis	Highexpression of lncRNA HOTAIR is an independent prognostic marker associated withworse metastasis-free survival, particularly inER+ BCpatients.
Zheng et al. 2019 [161]	P and M	160 BC and 60 normal	qRT-PCR	RHPN1-AS1 expression was upregulated in BC, and elevated RHPN1-AS1 expression was strongly associated with poor prognosis of BC patients.
Do et al. 2021 [62]	P	160 BC	Microarrays	LncRNAs were more informative than mRNAs in prediction of recurrence.
Beltrán-Anaya et al. 2019 [125]	P	156 BC	qRT-PCR	Low expression of LncKLHDC7B is associated with poor prognosis in BC patients.
Zheng et al. 2019 [162]	P	151 BC and 26 normal	qRT-PCR	LncRNA LUCAT1 might be a significant prognostic biomarker in BC.
Liu et al. 2015 [232]	P and M	139 BC and 28 normal	qRT-PCR	MiR-1 is downregulated in BC; patients with low miR-1 expression tend to have poorer survival.
Zhou et al. 2021 [88]	P	132 BC and 132 normal	qRT-PCR	RACGAP1P was upregulated in BC tissue, and its expression was positively correlated with LN, distancemetastasis, and TNM stage. RACGAP1P is a potential prognostic biomarker and therapeutic target for BC.
She et al. 2022 [48]	P	130 BC and 130 normal	qRT-PCR	MCF2L-AS1 was frequently highly expressed in BC tumors, and this overexpression was associated with worse patient outcomes. MCF2L-AS1 may represent a potential predictive biomarker and therapeutic target for BC.
Li et al. 2016 [218]	P	129 BC and 38 normal	qRT-PCR	MALAT1 was upregulated in TNBC compared with adjacent normal breast tissues (*n* = 38). High expression of MALAT1 in TNBC (*n* = 129) was correlated with late TNM stage and metastasis, but not with age and tumor size.
Sun et al. 2023 [38]	P	122 BC	qRT-PCR	The lncRNA COX10-DT might be a potential biomarker and therapeutic target for breast cancer.
Cui et al. 2020 [93]	P	120 BC	qRT-PCR	HOXA-AS2 expressions in samples from patients with Adriamycin-based neoadjuvant chemotherapy were significantly higher compared with samples from patients without neoadjuvant chemotherapy.
Dan et al. 2021 [61]	P	119 BC and 19 normal	qRT-PCR	Abundant Lnc-BTG3-7:1, which targets the gene C21ORF91, was specifically observed in TNBC biopsies. Lnc-BTG3-7:1 is a potential marker for prognosis and a therapeutic target for BC.
Chen et al. 2021 [58]	P	113 BC and 113 normal	qRT-PCR	Seven lncRNAs (ST8SIA6-AS1, lnc-HIST1H2BJ-5:1, lnc PRICKLE2-3:2, RP1-86C11.7, RP11-15F12.1, ZNF670-ZNF695 and lnc-STRN3-12:1) were significantly upregulated in BC. ST8SIA6-AS1 was associated with TNM staging and Ki-67 index.
Song et al. 2019 [149]	P	112 BC and 112 normal	qRT-PCR	LncRNA LINC01287 was correlated with poor clinical outcome and may function as a novel prognostic biomarker and therapeutic target in BC.
Safari et al. 2019 [146]	P	111 BC and 130 normal	ARMS-PCR	Genomic variants within the LncRNA H19 confer risk of breast cancer.
Shi et al. 2021 [78]	P	108 BC and 108 normal	qRT-PCR	High NORAD expression indicated a poor prognosis of BC patients.
Yang et al. 2016 [224]	P	102 BC and 95 normal	qRT-PCR	AFAP1-AS1 expression was 5-fold increased in TNBC compared with normal tissue and was associated with LN, distant metastasis, and stage, suggesting that AFAP1-AS1 may be a novel and a potential therapeutic target for TNBC.
Xiang et al. 2019 [154]	P	102 BC and 102 normal	qRT-PCR	Expression of lncRNA SPRY4-IT1 is an independent adverse prognostic factor for patients with BC.
Li et al. 2017 [199]	P and M	100 BC and 100 normal	qRT-PCR	LncRNA TUG1 is upregulated in BC and is associated with tumor size, distant metastasis and TNM staging.
Su and Hu 2017 [207]	P and M	100 BC and 100 normal	qRT-PCR	LncRNA HOXA11-AS is highly upregulated in BC and significantly correlated with tumor size, metastasis and TNM staging.

BC = Breast Cancer; DCIS = Ductal Carcinoma In Situ; lncRNA = long non-coding RNA; M = Metastatic BC; P = Primary BC; TNBC = Triple-Negative BC.

**Table 2:** Studies with non-coding RNA analyses in blood samples from breast cancer patients (in descending order of the number of blood samples).

Study	BC Stage	Number of Specimens	Type of Specimen	Primary Method	Main Findings and Conclusions
Lin et al. 2018 [175]	P	969 BC and 970 healthy	Whole blood	DNA genotyping	SNPs located in LncRNA HOTAIR (rs1899663 and rs7958904) are significantly associated with increased risk of BC.
Peng et al. 2018 [177]	P	487 BC and 489 healthy	Plasma	qRT-PCR	Genetic variants in LncRNAMALAT1, particularlyrs619586, are associated with BC susceptibility and influenceMALAT1mRNA expression.
Xu et al. 2017 [212]	P	439 BC and 439 healthy	Whole blood	MassArray	The rs3787016 TT genotype was associated with increased breast cancer risk and clinicopathologic features of the tumor, especially among premenopausal women.
Shin et al. 2019 [147]	DCIS, P and M	179 BC and 192 healthy	Whole blood	qRT-PCR	LncRNA NEAT1 confers oncogenic role in TNBC through modulating chemoresistance and cancer stemness.
Ali et al. 2020 [90]	P	150 BC, 95 benign, 154 healthy	Serum	qRT-PCR	Low serum levels of MEG3 protein andrs7158663 polymorphismofthe MEG3 geneare associated with an increasedrisk of breast cancerand serve asdiagnostic and unfavorable prognostic factors.
Shi et al. 2022 [49]	P	120 BC, 70 benign, 105 healthy	Serum	qRT-PCR	Serum exosomal lncRNA DANCR levels were significantly higher in BC patients, and thiswas an independent risk factor for BC.
Zhao et al. 2017 [214]	P and M	96 BC and 90 healthy	Plasma	qRT-PCR	Linc (large intergenic non-coding) RNA-ROR expression is significantly upregulated in BC, and its increased levels are associated with ER, PR, and lymph node metastasis.
Zhang Y et al. 2021 [86]	P	95 BC and 95 healthy	Plasma	qRT-PCR	The expression of plasma IGF2-AS was evidently downregulated in BC compared with that of healthy controls, suggesting that IGF2-AS may be a promising diagnostic and prognostic biomarker.
Zidan et al. 2018 [191]	P and M	80 BC and 80 healthy	Serum	qRT-PCR	LncRNA MALAT1 upregulation plays an essential role in the development of BC, and theserumlevelof MALAT1 could be a possible tumor marker for the diagnosis of breast cancer.
Miao et al. 2016 [220]	P	78 BC and 40 healthy	Serum	qRT-PCR	LncRNA’s MALAT1 upregulation plays an important role in BC development, and circulating MALAT1 level may be a potential tumor marker for BC diagnosis.
Choudrhy et al. 2021 [60]	P	36 obese vs. 33 non-obese BC	Whole blood	qRT-PCR	Obesity may have a role in inhibiting AP001429.1 expression, which may serve as a novel potential biomarker and therapeutic target for BC.
Wei et al. 2019 [153]	P	63 BC and 43 healthy	Plasma	qRT-PCR	Decreased plasma expression levels of lncRNA HAND2-AS1 were observed in TNBC patients compared with those in healthy controls.
Zhou, Gu et al. 2021 [87]	P	62 BC, 38 benign, 45 healthy	Serum	qRT-PCR	NEAT1 expression in serum extracellular vesicles (EVs) of BC patients was higher than that in benign individuals and healthy controls. NEAT1 expression in serum EVs was high and related to LN metastasis, PR, ER and Ki-67 in BC patients.
ShaghaghiTorkdari et al. 2024 [27]	P	58 BC and 50 healthy	Plasma	qRT-PCR	A low level of plasma TINCR could discriminate breast cancer patients from healthy control subjects.
El-Fattah et al. 2021 [64]	P	50 BC, 25 benign, 25 healthy	Serum	qRT-PCR	Serum levels of HOTAIR and PVT1 were significantly higher, NEAT1 was significantly lower in BC patients than controls; there was no significant difference for MALAT1.
Psathas et al. 2023 [36]	P	46 BC	Serum	qRT-PCR	Circulating HOTTIP may serve as a future prognostic biomarker for breast cancer patients.
Abdel-hamid et al. 2023 [31]	P	45 BC	Serum	qRT-PCR	Circulating HOTAIR and miR-130a may serve as non-invasive biomarkers for predicting the pathological features of breast cancer patients.
Oloomi et al. 2013 [240]	P	32 BC and 32 healthy	Serum	RT-PCR	The most released CK19 mRNAs in blood and tissue of breast cancer patients are ncRNA.
ElAlfy et al. 2026 [248]	P and M	21 P, 19 M, 10 healthy	Serum	qRT-PCR	Circulating PVT1, HOTAIR, miR-331, and miR-195 may serve as potential diagnostic and prognostic biomarkers for BC
Wang et al. 2019 [151]	DCIS, P and M	23 BC and 23 healthy	Plasma	qRT-PCR	LncRNA HOTAIR in circulatory exosomes is correlated with ErbB2/HER2 positivity in BC.

BC = Breast Cancer; DCIS = Ductal Carcinoma In Situ; lncRNA = long non-coding RNA; M = Metastatic BC; P = Primary BC; TNBC = Triple-Negative BC.

#### 2.3.2. Studies with Non-Coding RNA Analyses in Blood Samples from Breast Cancer Patients

Table 2 presents an overview of all studies with lncRNA analysis of blood samples from breast cancer patients. Six studies included more than 100 blood samples from breast cancer patients [49,90,147,175,177,212]. RNA was extracted from serum in 10 studies [31,36,49,64,87,90,191,220,240,248], from plasma in seven studies [28,60,147,151,153,175,212], and from whole blood in three studies [86,177,214]. The following non-coding RNAs were identified as potential diagnostic biomarkers and/or therapeutic targets in blood from breast cancer patients: HOTAIR [31,64,151,175], MALAT1 [177,191,220], rs3787016 TT [212], NEAT1 [64,87,147], MEG3 [90], DANCR [49], ROR [214], IGF2-AS [86], AP001429.1 [60], HAND2-AS1 [153], TINCR [28], PVT1 [64], and HOTTIP [36].

#### 2.3.3. Studies Involving Clinical Samples Complemented by In Vitro and Animal Experiments

Supplementary Table S2 presents 177 studies involving clinical samples from breast cancer patients complemented by additional in vitro analyses and 75 studies that included additional animal experiments. The most commonly used additional assays were the following: Cell culture, Cell counting kit-8 (CCK-8) assay, Cell growth assay, Cell invasion assay, Cell migration assay, Cell proliferation assays, Cell transfections, Cell viability assays, Chromatin immunoprecipitation (ChIP)-assay, Colony formation assay, Dual-luciferase reporter assay, 5-Ethynyl-2′-Deoxyuridine (EdU) Assay, Flow cytometry, Fluorescence In Situ Hybridization (FISH), Immunohistochemistry (IHC), RNA immunoprecipitation (RIP) assay, RNA pull-down, Transwell Assay, Western blot, and Wound Healing. Less commonly, the following additional assays were used: Cell phenotype assays, Annexin V apoptosis assay, Boyden assay, BrdU assay, Cell cycle analysis, DNA methylation analysis, ELISA, Gene strand-specific RT-PCR, Glucose metabolism, Glycolysis stress test, Immunofluorescence, Immunoblotting, In situ Hybridization (ISH), JC-1 assay, Laser capture microdissection, Mass spectrometry, Methylation-specific PCR, Microarray analysis, mRNA stability analysis, MTT-assay, Northern blot, 5′ and 3′ rapid amplification of cDNA ends (RACE), RNA-interference, triple-primer-PCR, TUNEL assay, and RNA-RNA in vitro interaction assay. Among these 177 studies that were complemented by additional in vitro analyses and 75 studies that included animal experiments, there were only six studies that analyzed at least 100 breast tumor tissue specimens with or without normal breast tissue samples [78,88,124,161,162,218]; the following lncRNAs were identified in these six studies as potential diagnostic biomarkers and/or therapeutic targets: NORAD [78], RACGAP1P [88], HUMT [124], RHPN1-AS1 [161], LUCAT1 [162], and MALAT1 [218].

## 3. Discussion

In recent years, long non-coding RNA (lncRNA) analyses have been widely conducted in pre-clinical and clinical breast cancer research in the hope of identifying new diagnostic biomarkers and potential therapeutic targets. However, the large volume of published studies in this area has blurred the big picture of existing knowledge and missing evidence, and the crucial question remains as to whether lncRNA analyses can be used in routine clinical practice at present or in the near future. Therefore, based on the commonly accepted guidelines upon secondary analysis of the published literature [19,20,249], we carried out the present scoping review in order to identify the types of available evidence in this area, to clarify key concepts in the literature, to examine how research is conducted, and to identify and analyze possible knowledge gaps [249].

Following a comprehensive search of the pertinent literature, we identified and included 230 clinically relevant studies [21,22,23,24,25,26,27,28,29,30,31,32,33,34,35,36,37,38,39,40,41,42,43,44,45,46,47,48,49,50,51,52,53,54,55,56,57,58,59,60,61,62,63,64,65,66,67,68,69,70,71,72,73,74,75,76,77,78,79,80,81,82,83,84,85,86,87,88,89,90,91,92,93,94,95,96,97,98,99,100,101,102,103,104,105,106,107,108,109,110,111,112,113,114,115,116,117,118,119,120,121,122,123,124,125,126,127,128,129,130,131,132,133,134,135,136,137,138,139,140,141,142,143,144,145,146,147,148,149,150,151,152,153,154,155,156,157,158,159,160,161,162,163,164,165,166,167,168,169,170,171,172,173,174,175,176,177,178,179,180,181,182,183,184,185,186,187,188,189,190,191,192,193,194,195,196,197,198,199,200,201,202,203,204,205,206,207,208,209,210,211,212,213,214,215,216,217,218,219,220,221,222,223,224,225,226,227,228,229,230,231,232,233,234,235,236,237,238,239,240,241,242,243,244,245,246,247,248]. After data extraction, data analysis, and the synthesis of the results, we found that most studies were carried out in specimens taken from patients with primary invasive breast cancer (stages I–III), as compared with metastatic disease (stage IV) and ductal carcinoma in situ (DCIS; stage 0). Therefore, the current knowledge regarding the possible role of lncRNAs in DCIS and metastatic breast cancer appears to be limited; future studies should focus on investigating the role of lncRNAs in DCIS and metastatic disease. It is worth noting that only a small proportion of studies included more than 100 tissue specimens (Table 1 and Table 2), suggesting that the results from most studies cannot be easily generalized. Hence, taking into account this limitation, future lncRNA analyses should include larger numbers of clinical specimens from breast cancer patients. In particular, as shown in Table 1, among the included studies of lncRNA analyses, the highest numbers of breast cancer tissue specimens were 5634 [84] and 1048 [241], and the highest numbers of blood samples from breast cancer patients were 969 [175] and 487 [177]; none of these studies had any direct effect on clinical practice. For comparison, clinical studies evaluating the prognostic and/or predictive value of gene signatures in early breast cancer included analyses from 10,273 patients for the 21-gene assay OncotypeDX [250], 6600 patients for the 70-gene assay Mammaprint [251], 2558 patients for the 50-gene assay PAM50 [252] and 2185 for the 12-gene assay Endopredict [253], and all of these assays are currently routinely used in clinical practice. Therefore, the maturity in the field of lncRNA analysis in breast tumor specimens should not be overestimated.

In the present scoping review, we also found that most lncRNA analyses ofclinical specimens were carried out in breast cancer tissue, with or without normal breast tissue (*n* = 231; Table 1 and Supplementary Table S1), and only a small proportion were carried out in blood samples from breast cancer patients (*n* = 21), i.e., 10 studies involving serum, seven studies involving plasma, and four studies involving whole blood (Table 2). Interestingly, unlike other molecular analytical approaches in breast cancer research, such as the use of proteomics [254], we did not identify any study with lncRNA analyses in other biologic materials such as nipple aspiration fluid (NAF), urine, saliva, tear fluid or pleural effusions. Although blood samples are more easily accessible than normal and cancerous breast tissue specimens, and despite the fact that blood samples may be obtained more than once during the course of disease, technical issues, especially those regarding RNA isolation and stability in blood samples [255], may explain this paucity of studies involving blood as compared with studies involving tissue samples from breast cancer patients. Therefore, given the knowledge gap in this direction, future research may focus on evaluating the possible role of lncRNAs in blood samples from breast cancer patients. In particular, bearing in mind the concept of tumor dormancy, which is firmly supported by the detection of circulating tumor cells (CTCs) and tumor cells in the bone marrow of breast cancer patients years before disease recurrence [3,4,5,6] and the most recent advances on the clinical validity and repeated CTC enumeration in metastatic breast cancer [256,257], delving deeper into the role of lncRNAs in blood samples from breast cancer patients seems to be a promising new frontier for translational research. However, current knowledge in this field should not be overestimated.

In the largest study to date in breast cancer tissue, Xu et al. [84] performed a two-stage genome-wide lncRNA association study onHan Chinese women, covering 5634 breast cancer patients and 6308 healthy controls, and identified a novel breast cancer-associated lncRNA, i.e., lncHSAT164, whichis implicated in regulating the cell cycle and may be a potential therapeutic target in breast cancer. Some years earlier, in a multicenter study, Redis et al. [241] analyzed the expression of CCAT2 in a set of 30 breast cancersamples from Romania, a tissue array containing 18 tumors from the USA, and an independent set of 997 primary breast cancers from the Netherlands, and concluded that the expressionof lncCCAT2 can predict clinical outcomes for a specific subgroup of breast cancer patients. In the largest study onblood samples, Lin et al. [175] assessed the interaction between three single-nucleotide polymorphisms (SNPs) of the lncRNA HOTAIR on the risk and clinical outcome of breast cancer in a Chinese Han population that included 969 breast cancer cases and 970 healthy controls and found that two SNPs (rs1899663 and rs7958904) were significantly associated with increased risk of breast cancer. In another large study, Peng et al. [177] assessed three SNPs of the lncRNA MALAT1 in a Chinese Han population that included 487 breast cancer cases and 489 cancer-free controls and found that the genetic variants of lncRNA MALAT1, particularlyrs619586, were associated with susceptibility to breast cancer. It is noteworthy that both HOTAIR and MALAT1 have been studied in multiple studies both in breast cancer tissue and blood from breast cancer patients, i.e., HOTAIR in six studies [31,64,151,175,239,248] and MALAT1 in 13 studies [33,64,66,76,121,177,191,215,216,218,220,226,232,247]. The role of these lncRNAs should not be overestimated, as these are the most extensively investigated or evaluated in the largest clinical cohorts, rather than fully validated clinical biomarkers. Other lncRNAs for which our literature search showed were evaluated in a relatively small number of specimens—for example, the female-specific lncRNA Xist [76,190,258]—may also deserve more attention in future studies.

Our comprehensive analysis of the literature also showed that lncRNA analyses of clinical samples from breast cancer patients were complemented by additional in vitro analyses in most of these studies (i.e., 177 out of 228, or 77.6% of the included studies). Furthermore, almost one-third of these studies (i.e., 75 out of 228, or 32.9% of the included studies) also included additional animal experiments (see Supplementary Table S2). Although these additional analyses reinforce the scientific basis and the validity of analyses of clinical specimens, still, their results cannot be directly translated into clinical applications. Therefore, future research should focus on analyzing lncRNAs in clinical samples of breast cancer patients, using the evidence already acquired from basic science approaches.

From a clinical perspective, among other breast cancer molecular subtypes, triple-negative tumors (i.e., tumors negative for estrogen receptors, progesterone receptors and HER2) have the poorest prognosis and limited treatment options. Therefore, it was not surprising that numerous studies on lncRNA analyses in clinical breast cancer specimens focused on triple-negative tumors [25,32,35,41,51,52,53,55,59,61,71,72,77,79,91,94,98,103,105,107,117,124,132,147,153,158,159,176,179,180,189,206,211,215,218,224,228], so that the role of lncRNA in triple-negative breast cancer could be further evaluated in a focused systematic review. On the other hand, studies on lncRNA in the neoadjuvant setting were scarce [93,110] despite the fact that in recent years, the administration of various combinations of neoadjuvant chemotherapy to breast cancer patients has been increasingly investigated in clinical trials. Hence, future studies should focus on evaluating possible clinical applications of lncRNAs in the neoadjuvant setting.

The main strength of the present scoping review is that this is the first comprehensive analysis of the literature regarding the current role of lncRNA in breast cancer diagnosis and treatment, providing an overview of research conducted in this area. Other published reviews on this topic were narrative reviews focusing on various issues, including the role of lncRNAs in the regulation of cancer stem cells and the tumor microenvironment [13,14], the cell cycle [15], inflammation in breast cancer [16], and the possibility to be used as therapeutic targets [17,18]. Therefore, the present review may be useful in designing primary studies investigating the possible role of lncRNAs as diagnostic and prognostic biomarkers and therapeutic targets for breast cancer patients. Furthermore, another strength of this scoping review is that we focused on studies analyzing lncRNA in clinical samples, i.e., in breast cancer tissue and/or blood from breast cancer patients, and also that we excluded studies conducted only in cell lines and in silico studies. With this approach, we provide a comprehensive outline of the current clinical role of lncRNAs in breast cancer. On the other hand, the exclusion of in vitro and in silico studies may be seen as a limitation, as some of these studies may well contain clinically relevant information. Another limitation of this scoping review lies in the search terms used, i.e., “non-coding RNA” and “ncRNA”, and the limit “title”. The use of more specific terms, such as “long non-coding RNA”, “lncRNA”, and “lincRNA”, as well as extending the search scope to the “Title/Abstract” field, might have yielded more relevant studies; this should be taken into account when designing relevant structured reviews in the future. It is worth noting that most studies (77.8%) included in the present scoping review were conducted in one country, namely China; this geographic concentration may limit the reproducibility, external validity and generalizability of findings across different populations due to existence of specific genotypes and specific healthcare system characteristics in a certain geographical area. Since this is a scoping review, in-depth analysis and the discussion of broader trends and several other aspects are beyond the scope of this article, and they would rather be the subject of focused systematic reviews; such aspects includethereproducibility of findings, especially in blood compared with tissue; consistency of the available evidence; extensive presentation of the remaining knowledge gaps in the field, findings from prospective vs. retrospective and single vs. multi-center studies; pooled Hazard Ratios; pooled sensitivity/specificity; survival analyses; Receiver-Operating Characteristic (ROC) comparisons for each lncRNA; comparisons of different primers used to detect the same lncRNA across studies; additional methods used for validation of findings; independent validation across multiple cohorts;the role of lncRNAs in different breast cancer subtypes and stages, especially in triple-negative and metastatic breast cancer; and the possible clinical utility of specific lncRNAs as prognostic and/or therapeutic biomarkers.

## 4. Materials and Methods

The present scoping review was carried out according to the Preferred Reporting Items for Systematic Reviews and Meta-analyses extension for Scoping Reviews (PRISMA-ScR) [19]; Supplementary Table S3 presents the PRISMA-ScR Checklist. The review protocol was registered in the Open Science Framework (registration DOI: 10.17605/OSF.IO/P3A).

### 4.1. Eligibility Criteria

The following population, concept, and context (PCC) criteria [19,20] were used: (1) Population: Patients diagnosed with breast cancer. (2) Concept: lncRNA analyses in breast cancer clinical research. (3) Context: Breast cancer and normal breast tissue, lymph nodes, serum, and/or plasma from breast cancer patients. Studies only concerningbreast cancer cell lines were not included.

The following inclusion criteria were applied: (1) Studies with lncRNA analyses of samples taken from breast cancer patients (in situ, primary and metastatic lesions, normal breast tissue, lymph nodes, serum, and plasma); (2) clinically relevant studies, with lncRNA analyses aiming at diagnostic and therapeutic target discovery and validation; (3) clinical studies. The following exclusion criteria were applied: (1) studies onmitochondrial ncRNA; (2) studies onother ncRNAs, including miRNAs and piRNAs; (3) in silico studies; (4) studies only onbreast cancer cell lines; (5) studies solely onanimal models; (6) reviews, comments, editorials, case series, case reports, and papers with specimens from fewer than 10 breast cancer patients analyzed; (7) retracted papers; and (8) papers in languages other than English.

### 4.2. Information Sources and Search of the Literature

This study was carried out by searching the two primary online medical databases: the PubMed/MEDLINE database (PubMed) and the EMBASE database (Scopus) for articles published until 14 April 2026. The following search terms were used in both database searches: “non-coding RNA” AND “breast cancer”, and “ncRNA” AND “breast cancer”, with the limit “title”.

### 4.3. Selection of Sources of Evidence

For study selection, two reviewers (PP and IG) screened independently all studies in order to cut out overlapping and duplicate studies and publications not written in English. The same two authors evaluated the titles and/or abstracts of the remaining articles based on the inclusion and exclusion criteria. Then, eligible studies were retrieved and the fulltexts were further evaluated in accordance with the inclusion and exclusion criteria. Conflicts were resolved by discussion or referral to a third reviewer (MZ), who also re-evaluated the selection of studies.

### 4.4. Data Charting Process, Data Items, and Synthesis of Results

Data collection was conducted independently by two authors (PP and IG) using Microsoft Excel. Conflicts were resolved by discussion or referral to a third reviewer (MZ), who also re-evaluated and confirmed collection of data. The following data were extracted from the included studies: first author; publication year; country of first author’s affiliation; clinical setting, i.e., if the possible clinical application was relevant to diagnosis or treatment; disease stage, i.e., ductal carcinoma in situ (DCIS, stage 0), primary invasive breast cancer (stages I, II and III), and metastatic breast cancer (stage IV); type of specimen analyzed, i.e., normal or tumor tissue, serum, or plasma; number of specimens analyzed; additional in vitro analyses; additional animal experiments; the primary method used in clinical samples; other methods used in clinical specimens, cell lines or animal samples; main conclusions. Extracted data were further analyzed according to clinical relevance, i.e., according to (a) the type of tissue specimen analyzed, (b) the number of specimens analyzed, and (c) the disease stage.

## 5. Conclusions

This is the first scoping review evaluating the current role of lncRNAs in clinically relevant breast cancer research. Our data analysis and synthesis of evidence showed that most lncRNA studies on clinical specimens were conducted in breast tumor specimens rather than blood samples from breast cancer patients. The most commonly used method was quantitative RT-PCR, and most studies were complemented by in vitro assays and almost one third by animal experiments. There were 39 studies that included at least 100 specimens from breast cancer patients, 33 studies on breast cancer tissue, and six studies on blood from breast cancer patients. A considerable number of studies focused on triple-negative breast cancer. The long non-coding RNAs HSAT164 and CCAT2 were identified in the two largest studies on breast cancer tissue and HOTAIR and MALAT1 in the two largest studies on blood, as clinically relevant. It should be noted, however, that these are not fully validated clinical biomarkers. At present, non-coding RNA analyses are not used routinely in clinical practice, but multiple studies on clinical specimens reinforced by results from in vitro and animal experiments suggest that various non-coding RNAs may be potential biomarkers for the diagnosis, disease monitoring, and treatment of breast cancer. However, since current supporting evidence comes only from observational studies supplemented by mechanistic laboratory experiments, the maturity of the field should not be overestimated.

## Data Availability

The original data presented in the study are openly available inMedline/PubMed (https://pubmed.ncbi.nlm.nih.gov/) and EMBASE/Scopus (https://www.elsevier.com/products/scopus), accessed on 14 April 2026.

## Supplementary Materials

The following supporting information can be downloaded at: https://www.mdpi.com/article/10.3390/epigenomes10040065/s1, Figure S1: A timeline chart presenting the number of studies of lncRNA in breast cancer published each year since 2009; Figure S2: Geographical distribution of published studies regarding lncRNA in breast cancer; Table S1: Studies with lncRNA analyses in less than 100 breast tumor specimens and/or normal breast tissue (in descending order of the number of specimens); Table S2: Studies with lncRNA analyses in specimens from breast cancer patents complemented by in vitro and animal experiments; Table S3: PRISMA-ScR Checklist.

## Abbreviations

The following abbreviations are used in this manuscript:

BC	Breast Cancer
CTCs	Circulating tumor cells
DCIS	Ductal Carcinoma In Situ
lncRNA	Long non-coding ribonucleic acid
PRISMA	Preferred Reporting Items for Systematic Reviews and Meta-analyses
qRT-PCR	Quantitative Reverse Transcription Polymerase Chain Reaction
ScR	Scoping Review
TNBC	Triple-Negative Breast Cancer
