# Supplementary material for: The Role of Long Non-Coding RNAs in Breast Cancer Diagnosis and Treatment Based on Evidence from Clinical Studies: A Scoping Review
Source: Epigenomes. 2026 Oct 7;10(4):65. doi: 10.3390/epigenomes10040065 (PMC13692496; doi:10.3390/epigenomes10040065)
Supplement: Supplementary file 1 [file epigenomes-10-00065-s001.zip › epigenomes-4563052-supplementary.pdf]

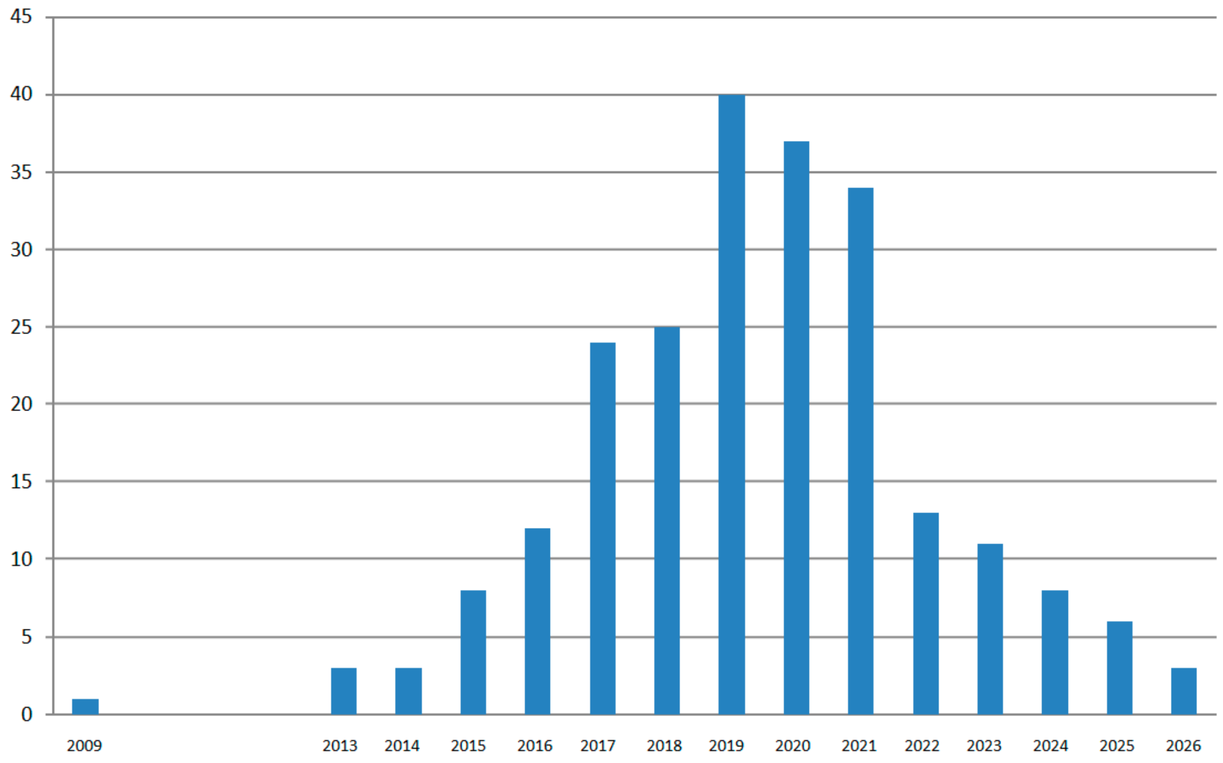

Supplementary Figure S1: A timeline chart presenting the number of studies of lncRNA in breast cancer published each year since 2009.

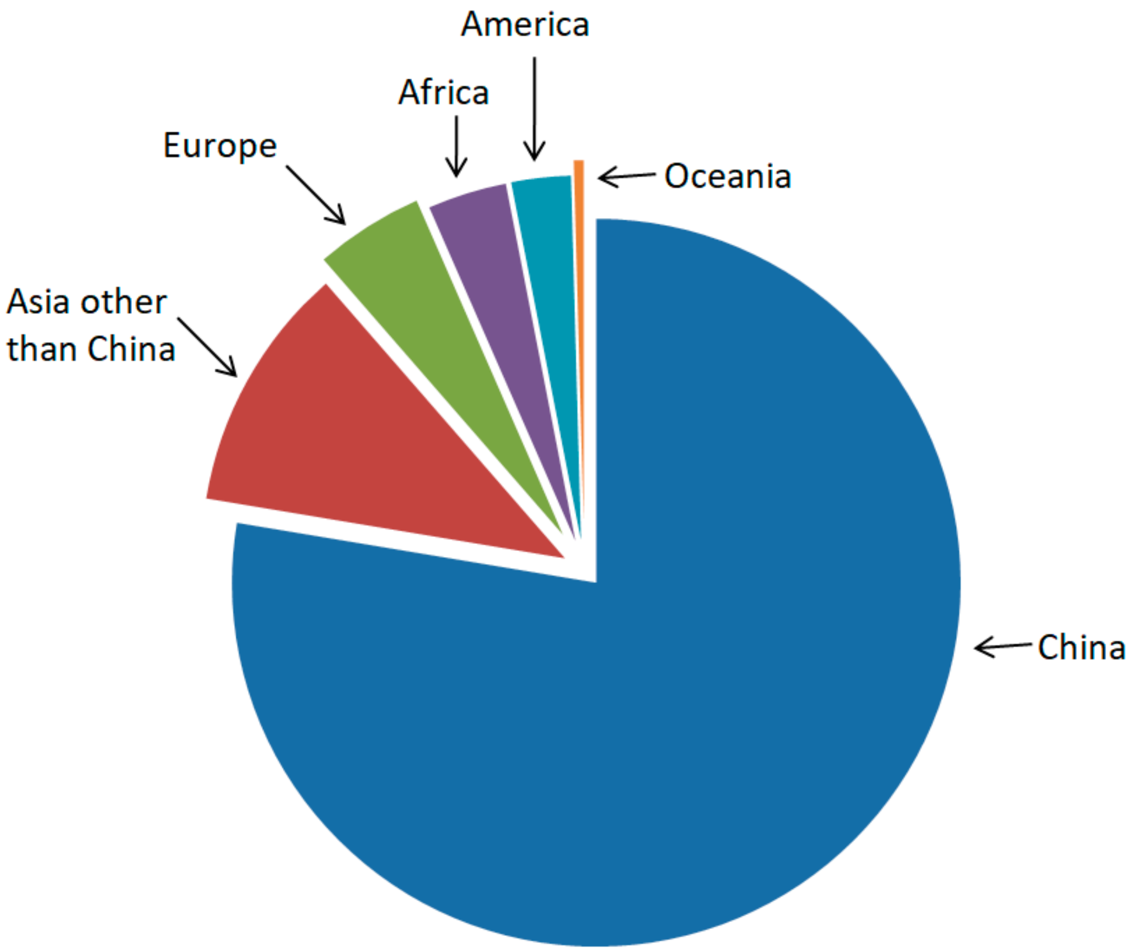

Supplementary Figure S2: Geographical distribution of published studies regarding lncRNA in breast cancer.

Supplementary Table S1: Studies with lncRNA analyses in less than 100 breast tumor specimens and/or normal breast tissue (in descending order of the number of specimens).

Study	BC stage	Number of BC specimens	Primary method	Main findings and conclusions
Guo et al. 2019 [132]	P	98 BC and 98 normal	qRT-PCR	LncRNA LINC00993 is breast-specific and is downregulated in breast cancer, especially in TNBC.
Zhang et al. 2019 [159]	P	98 BC and 98 normal	qRT-PCR	LncRNA ZEB2-AS1 promotes the proliferation, metastasis, and epithelial-mesenchymal transition (EMT) in TNBC through the PI3K/Akt/GSK3β/Zeb2 pathway.
Dong et al. 2018 [166]	P	98 BC and 36 normal	qRT-PCR	LncRNA SNHG14 promotes BC progression and chemo-resistance to trastuzumab treatment.
Wang et al. 2018 [181]	P	97 BC and 97 normal	qRT-PCR	LncRNA-CTD-2108O9.1 is downregulated in BC tissues and cells and acts as a metastatic inhibitor of BC by targeting leukemia inhibitory factor receptor (LIFR).
Yang et al. 2022 [52]	P	96 BC and 96 normal	qRT-PCR	Abnormal histone modification-mediated activation of LINC01559 enhances TNBC growth and metastasis. LINC01559 is a promising prognostic biomarket and therapeutic target for TNBC patients.
Zhang et al. 2022 [53]	P	95 BC and 95 normal	qRT-PCR	linc00921 was downregulated in TNBC. The linc00921/miR-9-5p/LZTS2 axis may be a novel biomarker and therapeutic target for TNBC patients.
Li et al. 2020 [105]	P	95 BC and 95 normal	qRT-PCR	LncRNA MNX1-AS1 was upregulated over 5-fold inTNBC and BC.
Zhang et al. 2015 [235]	P	92 BC and 92 normal	qRT-PCR	Up-regulation of lncRNA CCAT1 is correlated with aggressive disease progression and poor prognosis of BC patients.
Luo et al. 2023 [35]	P	91 BC	qRT-PCR	MIDEAS-AS1 is downregulated in TNBC tissues and low MIDEAS-AS1 levels predict poor prognosis of TNBC.
Li et al. 2019 [137]	P	89 BC and 89 normal	qRT-PCR	LINC01787 is up-regulated in BC and promotes cancer cell proliferation and migration, by binding to pre-miR-125b.
Yang et al. 2018 [184]	P	88 BC	qRT-PCR	LncRNA ARNILA is correlated with poor clinical outcome by modulating migration, invasion, EMT, metastasis and is negatively regulated by the androgen receptor.
Li et al. 2020 [108]	P	86 BC and 86 normal	qRT-PCR	SNHG1 expression was upregulated in 54 out of 86 BC clinical samples. SNHG1/miR-193a-5p/HOXA1 is a novel competing endogenous RNA regulatory pathway in BC progression and may provide new strategies for BC therapy.
Dong et al. 2019 [130]	P	85 BC and 85 normal	qRT-PCR	LncRNA LOXL1-AS1 upregulation facilitates BC invasion and metastasis by blocking miR-708-5p expression and activity.
Shen et al. 2021 [77]	P	84 BC and 84 normal	qRT-PCR	The expression level of YY1 was higher in TNBC tissues than in adjacent tissues. YY1 is a potential diagnostic and prognostic marker for the treatment of TNBC in the future.
Zhang et al. 2023 [41]	P	82 BC	qRT-PCR	PTCSC3 was downregulated, while lncRNA MIR100HG was upregulated in tumor tissues compared with adjacent non-cancerous tissues of patients with TNBC. Low levels of PTCSC3 and high levels of MIR100HG were associated with lower survival rates.
Ghafouri-Fard et al. 2020 [101]	P	82 BC and 82 normal	qRT-PCR	LncRNA HCP5 was significantly lower, while ITGB2-AS1 was higher in BC compared with normal breast tissue.

Supplementary Table S1 (continued – Part 2)

Ding et al. 2019 [129]	P	82 BC and 82 normal	qRT-PCR	LncRNA MIF-AS1 promotes breast cancer cell proliferation, migration and EMT process through regulating miR-1249-3p/HOXB8 axis.
Hashemi et al. 2025 [21]	P	80 BC and 80 normal	qRT-PCR	FAM83H-AS1 shows potential as a prognostic biomarker and therapeutic target in BC.
Yu et al. 2020 [121]	P	80 BC and 80 normal	qRT-PCR	MALAT1 was markedly elevated in tumor tissues versus adjacent tissues, and its expression was related to the clinical stage, lymph node metastasis, and tumor differentiation.
Yang et al. 2019 [157]	P & M	80 BC and 80 normal	qRT-PCR	LncRNA FGF14-AS2 binds to miR-205-5p to repress proliferation, migration, invasion, and induce apoptosis in BC.
Elias-Riszk et al. 2020 [97]	P	79 biopsies	qRT-PCR	lncRNA H19 could be a potential marker for BC diagnosis, prognosis and risk management.
Mehrpour Layeghi et al. 2020 [112]	P	79 BC and 79 normal	qRT-PCR	LINC00961 was significantly downregulated in luminal A and B BC tissues compared with adjacent non-tumoral tissues.
Miao et al. 2016 [220]	P	78 BC and 78 normal	qRT-PCR	LncRNA's MALAT1 upregulation plays an important role in BC development, and circulating MALAT1 level may be a potential tumor marker for BC diagnosis.
Huang et al. 2021 [66]	P	76 BC and 76 normal	qRT-PCR	MALAT1 expression was significantly increased in BC compared with adjacent normal tissue and it was significantly correlated with the Oncotype DX 21-gene results.
Xie et al. 2019 [155]	P	76 BC and 76 normal	qRT-PCR	LncRNA UFC1 regulates biological activity of breast cancer via miR-34a/CXCL10 axis.
Calanca et al. 2019 [126]	P	75 BC and 75 normal	qRT-PCR	Methylation status of isoforms of RASSF1 gene are cancer specific, while ANRASSF1 is differentially expressed between normal mammary cells and BC.
Li et al. 2017 [197]	P	74 BC and 74 normal	qRT-PCR	Inhibition on lncRNA ROR reverses resistance to tamoxifen by inducing autophagy in BC.
Liu et al. 2017 [203]	P	74 BC and 74 normal	qRT-PCR	LncRNA SUMO1P3 is overexpressed in BC and induces proliferation, migration, and invasion of BC cells by binding to and inhibiting miR-320a.
Kang et al. 2015 [231]	P	74 BC and 74 normal	qRT-PCR	LncRNA IRAIN is a novel imprinted gene that is aberrantly regulated in BC.
Pei et al. 2021 [73]	P	73 BC and 73 normal	qRT-PCR	RP11-283G6.5 low expression is related with poor prognosis.
Hu et al. 2019 [134]	P & M	72 BC and 72 normal	qRT-PCR	LncRNA BLACAT1 promotes BC proliferation and metastasis by regulating the miR-150-5p/CCR2 axis, suggesting it as a potential therapeutic target.
Chen et al. 2015 [229]	P & M	71 BC and 71 normal	qRT-PCR	Macrophages could promote invasiveness of BC cells by enhancing expression of lncRNA UCA1, which is upregulated in BC and associated with advanced clinical stage.
Fang et al. 2020 [98]	P	70 BC and 70 normal	qRT-PCR	SNHG22 was strongly expressed in TNBC. SNHG22 could serve as a promising molecular target for the development of targeted therapy in TNBC.
Zeng et al. 2018 [187]	P & M	70 BC and 70 normal	qRT-PCR	LncRNA OIP5-AS1 promotes the progression of BC by targeting SOX2 through miR-129-5p.
Xu et al. 2019 [156]	P	70 BC and 29 normal	qRT-PCR	LncRNA CBR3-AS1 is up-regulated in BC, and it is associated with prognosis of BC patients.

Supplementary Table S1 (continued – Part 3)

Yu et al. 2019 [158]	P & M	68 BC and 68 normal	qRT-PCR	LncRNA DRHC may serve as a tumor suppressor in TNBC and inhibit the proliferation of TNBC cells by downregulating lncRNA HOTAIR.
Li et al. 2017 [196]	P	68 BC and 68 normal	qRT-PCR	High expression of lncRNA HOXA11-AS promotes BC invasion and metastasis by affecting epithelial-mesenchymal transition.
Liu et al. 2017 [202]	P	65 BC and 65 normal	qRT-PCR	LncRNA OR3A4 is up-regulated in BC and its expression is associated with poor prognosis.
Mohamed et al. 2025 [245]	P	64 BC and 64 normal	qRT-PCR	LncRNAs (NKILA, LINC00993) levels in tumor tissue compared to normal tissue.
Li et al. 2018 [171]	P	64 BC and 64 normal	qRT-PCR	LncRNA NNT-AS1 promotes progression of BC through miR-142-3p/ZEB1 axis.
Yang et al. 2016 [225]	P	64 BC and 64 normal	qRT-PCR	LncRNA FGF14-AS2 is one of the down-regulated lncRNAs in BC and its expression is associated with patients' survival.
Wei et al. 2019 [153]	P	63 BC and 63 normal	qRT-PCR	LncRNA HAND2-AS1 may act as a tumor suppressor in TNBC by inhibiting cancer cell proliferation, through the downregulation of RUNX2.
Sha et al. 2017 [206]	P	63 BC and 63 normal	qRT-PCR	Knockdown of lncRNA DANCR inhibits proliferation, invasion and tumorigenesis of TNBC.
Li et al. 2020 [107]	P	62 BC and 62 normal	qRT-PCR	BAALC was significantly upregulated in TNBC compared with adjacent non-tumoral tissues. The LRRC75A-AS1/miR-380–3p/BAALC axis may be novel biomarkers for TNBC.
Behyari and Behyari 2024 [22]	P	62 BC and 62 normal	qRT-PCR	LncRNA LCAL4 was upregulated in BC compared with normal tissue. LncRNA LCAL4 is a potential new marker in the diagnosis of BC.
Li et al. 2019 [138]	P	62 BC and 62 normal	Microarray analysis	LncRNA ZFHX4-AS1 acts as an oncogene in BC via the Hippo signaling pathway by regulating FAT4.
Wang et al. 2022 [51]	P	60 BC and 60 normal	qRT-PCR	BRCAT54 was downregulated, while miR-130b was highly upregulated in TNBC tissues compared with non-tumor tissues
Escuin et al. 2024 [24]	P	60 primary and 60 SLN	RNA sequencing	Several sncRAs are potential surrogate markers of lymph node metastases and patient outcome in early-stage BC.
Liang et al. 2024 [26]	P	60 BC	qRT-PCR	Targeting the TMEM147-AS1/miR-124/STAT3 axis may be a target for ER-positive BC therapy
Ma et al. 2022 [45]	P	60 BC and 60 normal	qRT-PCR	Significantly higher DLEU1 expression was detected in tumors of advanced TNM stages,
Hu et al. 2021 [65]	P	60 BC and 60 normal	qRT-PCR	The expression of lncRNA ROR and TIMP3 was higher in BC tissues than that in the adjacent normal tissues. LncRNA ROR is a promising marker for prognosis and a therapeutic target for BC.
Huang et al. 2021 [67]	P	60 BC and 60 normal	qRT-PCR	TPT1-AS1 was downregulated in BC relative to the adjacent normal tissues. TPT1-AS1 ıs a promising tumor biomarker for patients with BCr.
Jiang et al. 2020 [102]	P	60 BC and 60 normal	qRT-PCR	Eight lncRNAs SNHG3, LNC00680, AC017048.4, MIR181A2HG, AC007461.2, LNC00277, GATA3-AS1 and AC017048.3 were upregulated in BC compared with normal breast tissue.
Lv et al. 2019 [141]	P & M	60 BC and 60 normal	qRT-PCR	LncRNA SNHG6 affects the cell proliferation and invasion partially through miR-26a-5p/MAPK6 pathways in the tumorigenesis of BC.

Supplementary Table S1 (continued – Part 4)

Ma et al. 2019 [142]	P	60 BC and 60 normal	qRT-PCR	LncRNA FGF13-AS1 acts as a tumor suppressor in BC by inhibiting glycolysis and stemness properties through an FGF13-AS1/IGF2BPs/Myc feedback loop.
Mehta-Mujoo et al. 2019 [143]	P	60 BC and 60 normal	qRT-PCR	LncRNA ANRIL is expressed in BC cells with varying subcellular localization, and its nuclear presence correlates with increased POSTN expression.
Wang et al. 2019 [152]	P & M	60 BC and 60 normal	qRT-PCR	LncRNA HULC is highly expressed in BC and promotes cancer proliferation, migration and invasion by sponging miR-6754-5p.
Song et al. 2018 [178]	P & M	60 BC and 60 normal	qRT-PCR	LncRNA GHET1 is up-regulated in BC tissues and cell lines, and promoted BC cell proliferation, invasion and migration by affecting EMT.
Chen et al. 2017 [192]	P	60 BC and 60 normal	qRT-PCR	LncRNA LINC00628 serves as a tumor suppressor in BC and is associated with regulation of Bcl-2/Bax/Caspase-3 signal pathway.
Wu et al. 2017 [209]	P	60 BC and 60 normal	qRT-PCR	LncRNA CCAT2 is highly expressed in BC and its downregulation inhibites the proliferation, invasion and migration of BC by regulatingTGFβ signaling pathway.
Fan et al. 2017 [193]	P & M	58 BC and 58 normal	qRT-PCR	Downregulation of the LncRNA TUG1 is associated with cell proliferation, migration, and invasion in BC.
Jiang et al. 2020 [103]	P	57 BC and 57 normal	qRT-PCR	DGCR5 was significantly upregulated in BDC tumor tissue samples than that in adjacent normal tissues. DGCR5 is a promising marker for TNBC.
Li et al. 2020 [104]	P	56 BC and 56 normal	qRT-PCR	Expression of lncRNA NEAT1 was upregulated significantly in human BC tissues compared with that in adjacent non-tumor tissues. Targeting lncRNA NEAT1 might be a promising approach to inhibit the growth and metastasis of BC cells.
Yang et al. 2018 [186]	P & M	56 BC and 56 normal	qRT-PCR	LncRNA-BCHE is oncogenic, enhances the growth and metastatic potential of BC cells.
Liu et al. 2015 [233]	P	55 BC and 55 normal	qRT-PCR	FOXCUT lncRNA may be functionally involved in the tumor progression of Basal Like BCs through the regulation of its paired mRNA, FOXC1.
Di et al. 2022 [42]	P	54 BC and 54 normal	qRT-PCR	The LncRNA MAFG-AS1 was upregulated in BC tissues, it was related to poor prognosis and it could be used as a diagnostic marker and therapeutic target
Liu et al. 2016 [219]	P & M	54 BC and 14 normal	qRT-PCR	Knockdown of lncRNA UCA1 inhibits the Wnt/β-catenin signaling and increases the tamoxifen sensitivity by promoting the apoptosis of BC cells when exposed to tamoxifen.
Li et al. 2021 [69]	P	52 BC and 52 normal	qRT-PCR	SOX21-AS1 was highly expressed in clinical BC compared with adjacent normal tissue. SOX21-AS1 is a potential prognostic biomarker for BC.
Gavgani et al. 2020 [100]	P	52 BC and 52 normal	qRT-PCR	HULC was upregulated in BC and may have a potential to be a new biomarker and therapeutic target in BC.
Guo et al. 2019 [133]	P & M	52 BC and 52 normal	qRT-PCR	LncRNA PRNCR1 is significantly upregulated in BC and is strongly associated with cancer progression and poor patient prognosis.
Ravanbakhsh Gavgani et al. 2019 [145]	P	52 BC and 52 normal	qRT-PCR	LncRNA USMycN is a potential molecular marker for BC and provides further evidence for critical role of this lncRNA in cancer.
Li et al. 2018 [173]	P	52 BC and 52 normal	qRT-PCR	LncRNA FENDRR inhibits cell proliferation and is associated with good prognosis in BC.

Supplementary Table S1 (continued – Part 5)

Cai et al. 2021 [56]	P	52 BC and 52 normal	qRT-PCR	The A1BG-AS1/miR-485-5p/FLOT1 pathway might offer a novel therapeutic perspective for BC.
Kia et a. 2026 [246]	P	51 BC and 51 normal	qRT-PCR	The expression of LINC01614 lncRNA in tumor tissues from breast cancer patients was 6.5 times higher than in adjacent normal tissues.
Bai et al. 2021 [55]	P	50 BC and 50 normal	qRT-PCR	SChLAP1 was upregulated and positively associated with the advanced progression of TNBC.
Han et al. 2024 [25]	P	50 BC and 50 normal	qRT-PCR	HANR expression was increased in TNBC, and this was correlated with disease progression in TNBC patients.
Hajibabaei et al. 2023 [33]	P	50 BC and 50 normal	qRT-PCR	The expression of MALAT1 and TOP2A in tumor tissues increased significantly in patients' tissues compared to normal tissue
Tan et al. 2023 [39]	P	50 BC and 50 normal	qRT-PCR	TINCR expression was downregulated in BC compared with normal tissue. TINCR was especially downregulated patients with large tumors, LN metastasis, and luminal subgroups A and B.
Tarighi et al. 2021 [80]	P	50 BC and 50 normal	qRT-PCR	LncRNA DSCAM-AS1 expression was significantly increased in BC compared with matched adjacent non-neoplastic tissues.
Gao et al. 2020 [99]	P	50 BC and 50 normal	qRT-PCR	RP11-480I12.5 expression was significantly upregulated in the BC compared with normal breast tissue.
Xiong et al. 2020 [119]	P	50 BC and 50 normal	qRT-PCR	SNHG1 expression was significantly higher in BC compared with normal breast tissues. SNHG1 could be a promising therapeutic target for patients with breast cancer.
Xi et al. 2017 [210]	P	50 BC and 50 normal	qRT-PCR	LncFOXO1 suppresses the growth of BC by recruiting BAP1 to the FOXO1 promoter.
Tarek et al. 2026 [247]	P	50 BC and 20 normal	qRT-PCR	MALAT1 was upregulated in BC tissue. miR-20a-5p, miR-135b were overexpressed whereas miR146a-5p levels were supressed.
Meng et al. 2024 [27]	P	48 BC and 48 normal	qRT-PCR	The lncRNA NCALD was overexpressed in luminal BC, which correlated with an unfavorable prognosis. Targeting lncRNA NCALD could be a potential therapeutic strategy.
Jiang et al. 2021 [68]	P	48 BC and 48 normal	qRT-PCR	lncRNA ANCR is down-regulated in BC and it can bind miR-331 and inhibit its expression to exert a tumor-suppressing effect.
Lian W et al. 2020 [109]	P	48 BC and 48 normal	qRT-PCR	LL22NC03-N64E9.1 expression was upregulated in the majority of BC samples (36 out of 48) compared with the adjacent noncancerous tissues. LL22NC03-N64E9.1 could be a therapeutic target for BC.
Jeong et al. 2018 [168]	P	48 BC and 48 normal	qRT-PCR	KLHDC7B and LncRNA STAR1 are both overexpressed in BC and significantly associated with gene modulation activity in the IFN signaling pathway during breast tumorigenesis.
Chen et al. 2015 [228]	P	48 BC and 48 normal	qRT-PCR	A set of lncRNAs that were consistently aberrantly expressed in TNBC, and notably LINC00993 was also associated with the expression of the estrogen receptor.
Li et al. 2018 [172]	P	48 BC and 47 normal	qRT-PCR	LncRNA LINC00310 promotes tumorigenesis by regulating c-Myc expression and it may determine prognosis of BC patients.
Sun et al. 2019 [150]	P	48 BC	qRT-PCR	The expression level of H19 was significantly associated with TNM stage, metastasis, and Ki67 index, but not with hormone receptor status, age, or histological grade. H19 could act as a potential predictive biomarker for HER2-positive BC, and its downregulation reverse trastuzumab resistance

Supplementary Table S1 (continued – Part 6)

Rezanejad and Asadi 2022 [46]	P	47 BC and 47 normal	qRT-PCR	VIM-AS1 was expressed 32% of breast tumors (15 out of 47) and upregulated in high-grade breast tumor tissues. VIM-AS1 is a potential diagnostic and prognostic BC.
Sarrafzadeh et al. 2017 [205]	P	47 BC and 47 normal	qRT-PCR	GHET1 was up-regulated in 26% (13 out of 47) BC compared with normal breast tissue, and may play a role in the pathogenesis of a fraction of BC tumors.
Cheng L et al. 2020 [92]	P	47 BC and 20 normal	qRT-PCR	LINC00662 was significantly upregu lated in BC tissues compared to the adjacent normal tissue. Targeting LINC00662 may represent a novel strategy for breast cancer therapy.
Liu et al. 2021 [70]	P	46 BC and 46 normal	qRT-PCR	Low expression of NORAD indicates poor prognosis for patients with BC.
Zhang et al. 2020 [122]	P & M	46 BC and 46 normal	qRT-PCR	LncRNA DANCR promotes BC progression by enhancing cancer stemness, epithelial-mesenchymal transition, and inflammation through EZH2-mediated suppression of SOCS3.
Zhao et al. 2019 [160]	P	46 BC and 46 normal	qRT-PCR	LncRNA HEIH may contribute to BC development via modulation of microRNA-200b/axis and inducing the activation of Wnt/β-catenin pathway.
Song et al. 2025 [176]	P	46 BC and 46 normal	qRT-PCR	circGNAI2 expression was negatively associated with lymph node metastasis and TNM tumor stage
Torkashvand et al. 2021 [81]	P	45 BC and 45 normal	qRT-PCR	CBR3-AS1, RAB6C-AS1 and ZEB2-AS1 were upregulated in BC in comparison with normal tissue. Only CBR3-AS1 lncRNA has a high chance to be a BC biomarker.
Wang et al. 2020 [118]	P	45 BC and 45 normal	qRT-PCR	LINC00324 was significantly lower in BC compared to adjacent normal tissues. LINC00324 may be an early diagnostic marker and a novel therapeutic agent for BC.
Keshavarz and Asadi 2019 [136]	P & M	45 BC and 45 normal	qRT-PCR	LncRNA ES1 controls the proliferation of BC by regulating the Oct4/Sox2/miR-302 axis.
Li et al. 2017 [198]	P	45 BC and 45 normal	qRT-PCR	LncRNA H19 promotes proliferation and invasion of BC through the miR-152/DNMT1 axis.
Chen et al. 2021 [59]	P	45 BC and 45 normal	qRT-PCR	CCNE1 expressed highest level in TNBC compared with luminal A BC and normal breast tissue. The SENP3-EIF4A1-miR-195-5p-CCNE1 axis promotes TNBC progression.
De Palma et al. 2020 [96]	P	45 BC and 16 normal	qRT-PCR	LINC01087 was significantly downregulated in TNBC and upregulated in luminal BC subtypes in comparison to normal breast tissue.
Wu et al. 2021 [83]	P	44 BC and 44 normal	qRT-PCR	LINC01094 expression was markedly higher in BC tissues in comparison to para-cancerous tissues.
Mathias et al. 2021 [72]	P	44 BC	qRT-PCR	NORAD is downregulated, while HCG11 is upregulated in TNBC compared with luminal A tumors.
Shekher et al. 2023 [37]	P	43 BC and 43 normal	qRT-PCR	H19, MEG3 and GAS5 were non-significantly upregulated in BC tissue in comparison to normal adjacent tissue and significantly up- or down-regulated in different BC subgroups.
Wang et al. 2020 [117]	P	43 BC and 43 normal	qRT-PCR	THOR was over expressed in BC tissues, especially in TNBC. THOR may be a key lncRNA molecule regulating TNBC occurrence and development.
Qian et al. 2017 [204]	P	43 BC and 43 normal	qRT-PCR	LncRNA NEAT1 might be an oncogenic lncRNA that promotes proliferation of BC through the NEAT1/miR-101/EZH2 axis.
Zuo et al. 2017 [215]	P & M	43 BC and 43 normal	qRT-PCR	LncRNA MALAT1 promotes proliferation and invasion via targeting miR-129-5p in TNBC.

Supplementary Table S1 (continued – Part 7)

Chang et al. 2021 [57]	P	42 BC	qRT-PCR	CASC9/miR-590-3p/SIX1/NF-κB axis is involved in BC progression. CASC9 may represent an attractive therapeutic target for the prevention and treatment of BC.
Vennin et al. 2017 [208]	P	41 BC	qRT-PCR	LncRNA 91H is overexpressed in BC and it induces epigenetic modifications on H19/IGF2 locus.
Samir et al. 2021 [76]	P	41 BC and 41 normal	qRT-PCR	PD-L1 and MALAT1 expression was higher in all BC subtype tissues compared with healthy tissues, but not in lymph nodes; miR-182-5p was upregulated in both and XIST was downregulated in both.
Du et al. 2020 [96]	P	40 BC and 40 normal	qRT-PCR	LINC01705c expression was upregulated in BC tissues relative to normal adjacent tissues. LINC01705 is implicated in the progression of BC.
Du et al. 2021 [63]	P	40 BC and 40 normal	qRT-PCR	VCAN-AS1 is upregulated in BC and promotes its progression by regulating the miR-106a-5p-mediated STAT3/HIF-1α pathway. VCAN-AS1 may serve as a potential target for BC therapy
Qin et al. 2021 [74]	P	40 BC and 40 normal	qRT-PCR	High concentrations of cholesterol were found in lnc030-high breast tumors in comparison with lnc030-low tumors. The lnc030-SQLE-cholesterol synthesis pathway may serve as an effective therapeutic target for BC.
Si et al. 2019 [148]	P & M	40 BC and 40 normal	qRT-PCR	LncRNA H19 is upregulated in BC and promotes tumor growth, invasion, and migration while inhibiting apoptosis by regulating miR-138 and SOX4.
Zheng et al. 2018 [190]	P & M	40 BC and 40 normal	qRT-PCR	LncRNA XIST is down-regulated in BC and inhibits cell growth, migration, and invasion via the miR-155/CDX1 axis.
Fan et al. 2025 [243]	P	40 BC and 40 normal	qRT-PCR	LIMD1- AS1 expression is correlated with poor clinical outcomes in patients with breast cancer.
Zhu et al. 2021 [89]	P	40 BC and 15 normal	qRT-PCR	TMPO-AS1 expression was markedly upregulated in BC compared with adjacent non-cancerous tissues. TMPO-AS1 may serve as a potential therapeutic target in patients with BC
Zhou et al. 2019 [164]	P	39 BC, 10 hy-perplasia, 10 normal	qRT-PCR	LncRNA-NORAD promotes proliferation, invasion and migration of BC cells, correlates with worse prognosis and activates TGF-β/RUNX2 signaling pathway.
Bhandari et al. 2024 [23]	P	39 BC and 39 normal	qRT-PCR	LINC00651 may be a new marker and therapeutic target for BC.
Li et al. 2020 [106]	P	39 BC and 39 normal	qRT-PCR	GAS5 is downregulated in BC clinical samples compared with that in adjacent non-tumor tissues. The results demonstrated that GAS5 may not be a promising indicator for predicting chemosensitivity in BC.
Selezneva et al. 2022 [47]	P	38 BC and 38 normal	qRT-PCR	Methylation of the lncRNA genes MEG3, SEMA3B-AS1, and ZNF667-AS1 is involved in both the occurrence and progression of BC. Methylation of the GAS5 gene was detected only in the late stages of BC.
Liu et al. 2019 [140]	P	38 BC and 38 normal	qRT-PCR	LncRNA TUBA4B is downregulated in BC and functions as a tumor suppressor by inhibiting proliferation and invasion through directly targeting miR-19.
Fang et al. 2017 [194]	P & M	38 BC and 38 normal	qRT-PCR	LncRNA HOXA-AS2 is up-regulated in BC, its expression is associated with cancer agressiveness and it acts as a molecular sponge for miR-520c-3p and regulates its targets.
Azadeh et al. 2021 [54]	P	37 BC and 37 normal	qRT-PCR	LncRNA CCAT1 was downregulated in BC tissue compared with normal tissue. LncRNA CCAT1 could be considered as a potential diagnostic biomarker for BC.
Wang et al. 2020 [114]	P	37 BC and 37 normal	qRT-PCR	ROR expression was significantly upregulated in the BC compared with normal breast tissue.

Supplementary Table S1 (continued – Part 8)

Xu et al. 2017 [211]	P & M	37 BC and 37 normal	qRT-PCR	LncRNA ANRIL overexpression modulated TNBC tumorigenesis through acting as molecular "sponge" for miR-199a.
Li et al. 2019 [139]	P	35 BC and 35 normal	qRT-PCR	LncRNA UCA1 upregulates PTP1B expression by sequestering miR-206 at the post-transcriptional level, leading to enhanced BC cell proliferation and tumor growth.
Gao et al. 2018 [167]	P	35 BC and 35 normal	qRT-PCR	LncRNA CASC2 inhibits BC cell growth and metastasis through the regulation of the miR-96-5p/SYVN1 pathway.
Xia et al. 2018 [183]	P & M	35 BC and 35 normal	qRT-PCR	LncRNA LINC00673 promotes BC proliferation and metastasis through regulating B7-H6 and epithelial-mesenchymal transition.
Jiang et al. 2019 [135]	P	34 BC and 34 normal	qRT-PCR	LncRNA FOXD2-AS1 is involved in BC proliferation, invasion, migration and stemness through FOXD2-AS1/miR-150-5p/PFN2 axis.
Zhao et al. 2020 [125]	P & M	32 BC and 32 normal	qRT-PCR	LncRNA TUSC8 and MYLIP protein might serve as promising diagnostic biomarkers for breast cancer.
Oloomi et al. 2013 [240]	P	32 BC and 32 normal	RT-PCR	The most released CK19 mRNAs in blood and tissue of BC patients are non-coding RNA.
Cooper et al. 2009 [242]	P	32 BC	qRT-PCR	Non-coding SRA was differentially expressed in samples with high than low PR, while there was no difference with ER.
Wang et al. 2018 [182]	P	30 BC	qRT-PCR	LncRNA FTH1P3 activates paclitaxel resistance in BC through miR-206/ABCB1.
Liu et al. 2020 [110]	P	30 NACT resistant vs. 30 NACT-sensitive	qRT-PCR	The expression of UCA1 was considerably higher in NACT-resistant than NACT-sensitive BC tissue (NACT-resistant was progressive disease during NACT or recurrent disease within 6 months of completing NACT). UCA1 might be a significant therapeutic biomarker.
Zarei et al. 2023 [40]	P	30 BC and 30 normal	qRT-PCR	ZEB2-AS1 expression is significantly upregulated in tumor tissues, especially in advanced stages and ZEB2-AS1 is associated with the aggressiveness of tumors by functioning as putative oncogenic lncRNA
Li et al. 2022 [44]	P	30 BC and 30 normal	qRT-PCR	PVT1 was upregulated and facilitated cell migration and invasion of BC by regulation of miR-148a-3p and ROCK1, indicating that PVT1 may be a potential biomarker of BC diagnosis and treatment.
Tang et al. 2022 [50]	P	30 BC and 30 normal	qRT-PCR	The PCAT1/miR-134-3p/PITX2 axis could be a promising therapeutic target in BC treatment.
Sun et al. 2021 [79]	P	30 BC and 30 normal	qRT-PCR	TTN-AS1 expression was significantly increased, whereas the expression of miR-211-5p was significantly decreased in TNBC tissues compared with adjacent tissues. TTN-AS1 may be considered as a significant prognostic biomarker.
Ding et al. 2020 [95]	P	30 BC and 30 normal	qRT-PCR	The expression of MAFG-AS1 was increased in tumor tissues relative to adjacent normal tissues. MAFG-AS1 is a promising therapeutic approach for BC.
Yeganeh et al. 2020 [120]	P	30 BC and 30 normal	qRT-PCR	The levels of four lncRNAs NeST, NTT, HULC and BACE1 were increased in the BC compared with normal tissue. Therefore, these four lncRNAs is a potential biomarker panel for BC prognosis.

Supplementary Table S1 (continued – Part 9)

Cao et al. 2019 [127]	P	30 BC and 30 normal	qRT-PCR	LncRNA UASR1 is upregulated in BC tissues and promotes BC cell growth, proliferation, wound healing and migration through activation of the AKT/mTOR signaling pathway.
Gao et al. 2019 [131]	P	30 BC and 30 normal	qRT-PCR	SNHG7 is upregulated in BC and acts as an oncogene to promote cell proliferation and invasion by directly sponging miRNA-381.
Zhou et al. 2019 [163]	P & M	30 BC and 30 normal	qRT-PCR	LncRNA TFAP2A-AS1 functions as a tumor suppressor in BC by inhibiting cell proliferation and invasion through the miR-933/SMAD2 axis.
Zhu et al. 2019 [165]	P	30 BC and 30 normal	qRT-PCR	c-Myc induces the regulation of LncRNA RHPN1-AS1 on BC cell proliferation via inhibiting P53.
Yang et al. 2018 [185]	P	30 BC and 30 normal	qRT-PCR	LncRNA MAGI2-AS3 is decreased in BC and promotes Fas and FasL protein expression, thereby inhibiting BC proliferation.
Zhang et al. 2018 [188]	P	30 BC and 30 normal	qRT-PCR	LncRNA FEZF1-AS is up-regulated in BC and is involved in FEZF1-AS1/miR-30a/Nanog axis, regulating BC stemness and tumorigenesis.
Zhang et al. 2018 [189]	P	30 BC and 30 normal	qRT-PCR	LncRNA HOST2 is involved in the proliferation of TNBC via regulation of the let-7b/CDK6 axis.
He et al. 2015 [230]	P	30 BC and 30 normal	qRT-PCR	Upregulated lncRNA-AK058003 in BC cells promotes BC cell proliferation, invasion and metastasis via the regulation of SNCG expression.
Al-Hazani et al. 2023 [32]	P	29 BC	qRT-PCR	SNHG11 was up-regulated and miR-7–5p down-regulated in clinical TNBC tissues.
Xue et al. 2021 [85]	P	282 BC	qRT-PCR	Patients with lower Linc01315 expression level had significantly longer disease-free and overall survival. Linc01315 may represent an independent prognostic marker and therapeutic target in BC.
Qin et al. 2021 [75]	P	28 BC and 28 normal	qRT-PCR	CDKN2B-AS1 is upregulated in BC compared with normal tissue. lncRNA CDKN2B-AS1 acts as a miR-122-5p sponge to regulate the STK39 expression, and promotes breast cancer progression.
Sun et al. 2020 [113]	P	26 positive SLN and 20 negative SLN	qRT-PCR	Lnc-ANGPTL1-3:3, lnc-GJA10-12:1, lnc-ACAN-2:1 and lnc-ZPBP2-4:1 were significantly downregulated in the SLN (+) compared with SLN (-) groups with PCR, but the the first two also in silico. Lnc-GATA3-16:1 and lnc-ACOX3-5:1 did not exhibit significant differential expression levels with PCR
Liu et al. 2020 [111]	P	26 BC and 26 normal	qRT-PCR	A2M-AS1 and MUC19 were highly expressed in BC, while miR-146b was poorly expressed. The result could open a new treatment target in BC treatment field.
Chen et al. 2019 [128]	P	26 BC and 26 controls	qRT-PCR	LncRNA LINC00312 may regulate BC progression as a tumor-suppressor gene, while LINC00312/miR-9/CDH1 axis contributes to the progression of BC.
Reiche et al. 2014 [237]	P	26 BC and 26 controls	Microarray analysis	A number of differentially expressed lncRNAs have been identified with relation to cancer-related protein-coding genes.
Wang et al. 2021 [82]	P	26 BC and 26 controls	qRT-PCR	CASC7 was significantly down-regulated in BC, and boosting the expression level of CASC7 retarded BC progression.
Yi et al. 2024 [29]	P	25 BC and 25 normal	qRT-PCR	lncRNA MEG3 and CNN1 were upregulated in BC tissues and cells, while miR-330 was downregulated.
Tao et al. 2018 [180]	P	25 BC and 25 normal	qRT-PCR	Knocking down the LncRNA TP73-AS1 suppresses vasculogenic mimicry in TNBC cells by targeting the miR-490-3p/TWIST1 axis.

Supplementary Table S1 (continued – Part 10)

Wang et al. 2020 [116]	P & M	24 BC and 24 normal	RT-PCR	LncRNA GACAT1 can adsorb microRNA-875-3p to upregulate the level of STON2, thereby promoting the progression of BC.
Zhao et al. 2017 [214]	P & M	24 BC and 24 normal	qRT-PCR	Linc (Large intergenic non coding) RNA-ROR expression is significantly upregulated in BC and its increased levels are associated with ER, PR, and lymph node metastasis.
Chen et al. 2020 [91]	P	20 BC and 20 normal	qRT-PCR	Increased MIR100HG expression was observed in TNBC tissues, whereas that of miR-5590-3p was downregulated. MIR100HG is a potential therapeutic target for BC.
Huang et al. 2023 [34]	P	20 BC and 20 normal	qRT-PCR	LINC00052 expression was significantly upregulated in BC tissues compared with adjacent normal breast tissue
Li et al. 2022 [43]	P	20 BC and 20 normal	qRT-PCR	LINC00092 is a methylation-driven lncRNA in BC and could be a potential therapeutic target. LINC00092 RNA-expression was lower and DNA methylation was higher in BC compared with normal breast tissue.
Wang et al. 2020 [115]	P	20 BC and 20 normal	qRT-PCR	H19 was highly expressed in BC tumor tissues compared with normal tissues. H19 might provide an effective target for the treatment of BC.
Pang et al. 2019 [144]	P & M	20 BC and 20 normal	qRT-PCR	LncRNA PRNCR1-2 is highly expressed in BC tissues, and its depletion results in the suppression of cell proliferation, migration, invasion and cell cycle progression.
Tang et al. 2018 [179]	P	20 BC and 20 normal	qRT-PCR	LncRNA TUG1 is downregulated and functions as a chemotherapy sensitizer in TNBC, while crosstalk among TUG1, miR-197 and NLK plays a key role in mediating chemotherapy response.
Zhang et al. 2017 [213]	P	20 BC and 20 normal	qRT-PCR	LncRNA RPPH1 functions as a tumour promoter and plays an important role in advancing tumorigenesis by targeting miR-122 in BC.
Sun et al. 2016 [221]	P	20 BC and 20 normal	qRT-PCR	LncRNA MEG3 is downregulated in BC tissues and affects BC cells' malignant behaviors, stabilizing and accumulating p53.
Yang et al. 2016 [226]	P	20 BC and 20 normal	qRT-PCR	MALAT1 was significant downregulated in BC compared with normal breast tissues.
Ding et al. 2014 [236]	P	20 BC and 20 normal	qRT-PCR	Four selected lincRNAs (BC2,BC4, BC5, and BC8) were aberrantly expressed in BC.
Shi et al. 2014 [238]	P	20 BC and 20 normal	qRT-PCR	LncRNA Loc554202 is increased in BC, is associated with advanced pathologic stage and its knockdown induces apoptosis and inhibits migration/invasion.
Huang et al. 2025 [244]	P & M	20 BC and 20 normal		
Zhuang et al. 2024 [30]	P	197 BC (tissue array)	RNAscope	Patients with positive lncRNA TCONS_I2_00001955 expression had a shorter median disease-free survival than those with negative expression
Liang et al. 2018 [174]	P & M	18 BC and 18 normal	qRT-PCR	LncRNA is overexpressed in BC and can regulate its progression and chemoresistance via modulating the expression levels of miR-4766-5p and SIRT1.
Wu et al. 2016 [222]	P	18 BC and 18 normal	RNA sequencing	516 pairs of lncRNA-mRNAs have differential co-expression pattern (correlation between lncRNA and mRNA differs in BC and normal tissue); 291 pairs have dose-response co-expression pattern (similar correlation, but the expression level of lncRNA or mRNA differs in BC and normal.
Marcu et al. 2021 [71]	P	18 BC (9 TNBC and 9 luminal A)	qRT-PCR	GAS5 lncRNA has differential expression in TNBC tissues and cells compared with luminal A, with possible implications in the molecular mechanisms of the TNBC subtype.

BC = Breast Cancer; DCIS = Ductal Carcinoma In Situ; M = Metastatic BC; NACT = Neoadjuvant chemotherapy; P = Primary BC; qRT-PCR = quantitative reverse transcription polymerase chain reaction; SLN = Sentinel Lymph Node; TNBC = Triple Negative BC.

Supplementary Table S2: Studies with lncRNA analyses in specimens from breast cancer patents complemented by in vitro and animal experiments

Study	Type of sample	Cell lines	Animal experiments	Primary method	Other methods used
Han et al. 2024 [25]	BC and normal tissue	Yes	Yes	qRT-PCR	Cell growth assay, Western blot, RNA pull-down, RNA immunoprecipitation assay, Mass spectrometry analysis, Glucose metabolism, Glycolysis stress test.
Meng et al. 2024 [27]	BC and normal tissue	Yes	Yes	qRT-PCR	Western blot, ISH, FISH, RNAi and cell transfection, Flow cytometry, Annexin V apoptosis assay, Dual-luciferase reporter assay, RNA pulldown assay, RNA immunoprecipitation.
Huang et al. 2023 [34]	BC and normal tissue	Yes	Yes	qRT-PCR	IHC, ISH, RNA pull down, Dual-Luciferase Reporter Assay, Wound Healing and Transwell Assays, Western blot.
Luo et al. 2023 [35]	BC tissue	Yes	Yes	qRT-PCR	Cell migration and invasion assay, FISH, Chromatin immunoprecipitation (ChIP)-qPCR assay, RNA pull-down, Western blot, Cell proliferation assay, Colony formation assay.
Di et al. 2022 [42]	BC and normal tissue	Yes	Yes	qRT-PCR	RNA interference, Cell-proliferation assays, Cell migration and invasion assays, Flow-cytometry.
Ma et al. 2022 [45]	BC and normal tissue	Yes	Yes	qRT-PCR	Colony formation assay, Transwell assay, RNA immunoprecipitation (RIP), RNA pull-down assay, Chromatin immunoprecipitation (ChIP) assays.
Yang et al. 2022 [52]	BC and normal tissue	Yes	Yes	qRT-PCR	Chromatin immunoprecipitation (ChIP) assay, Wound healing and Transwell assays, RNA pull-down and immunoprecipitation (RIP) assays, Luciferase reporter assay.
Zhang et al. 2022 [53]	BC and normal tissue	Yes	Yes	qRT-PCR	Western blot, IHC, Cell counting kit-8 (CCK-8) assay, Wound-healing experiments, Transwell assay, FISH, Dual-luciferase reporter assay, RNA immunoprecipitation (RIP).
Cai et al. 2021 [56]	BC tissue	Yes	Yes	qRT-PCR	Cell Counting Kit-8 assay, Flow cytometry, Transwell migration and invasion assays, IHC, RNA immunoprecipitation assay, Luciferase report assay, Western blot.
Chen et al. 2021 [59]	BC tissue	Yes	Yes	qRT-PCR	Western blot, RNA Immunoprecipitation (RIP), Dual Luciferase reporter assay, Cell Proliferation and Cell Cycle Assays.
Du et al. 2021 [63]	BC and normal tissue	Yes	Yes	qRT-PCR	Western blot, Transwell assay, Detection of cell proliferation and viability, Colony formation assay, Dual luciferase activity assay, RIP assay, Flow cytometry.
Hu et al. 2021 [65]	BC and normal tissue	Yes	Yes	qRT-PCR	FISH, RNA pull-down assay, RNA pull-down assay, RIP assay, Chromatin immunoprecipitation (ChIP), Western blot, 5-Ethynyl-2′-deoxyuridine (EdU) staining, Transwell assay, Fow cytometry.
Pei et al. 2021 [73]	BC and normal tissue	Yes	Yes	qRT-PCR	Cellular growth and apoptosis assays, Transwell migration and invasion assays, IHC, Western blot, RNA–protein interaction assays, Dual-luciferase reporter assays, Immunofluorescence.
Qin et al. 2021 [74]	BC and normal tissue	Yes	Yes	qRT-PCR	Mammosphere Formation Assay, Western blot, IHC, FISH, Flow cytometry, Colony-Formation Assay, Total Cholesterol Concentrations and ALDH Activity Assay, RIP and RNA pull-down assay.
Qin et al. 2021 [75]	BC and normal tissue	Yes	Yes	qRT-PCR	Colony formation assays, luciferase reporter assay, Western blot, IHC.
Shen et al. 2021 [77]	BC and normal tissue	Yes	Yes	qRT-PCR	Western blot, Methylation-specific PCR (MSP), Chromatin immunoprecipitation (ChIP) assay, Cell counting kit-8 (CCK-8) assay, Transwell assay IHC.

Supplementary Table S2 (continued – Part 2)

Study	Type of sample	Cell lines	Animal experiments	Primary method	Other methods used
Shi et al. 2021 [78]	BC and normal tissue	Yes	Yes	qRT-PCR	MTT assay, Colony formation assay, Transwell assay, Western blot, TUNEL assay, IHC, RNA pull-down assay, RIP assay, Dual luciferase reporter gene assay.
Wu et al. 2021 [83]	BC and normal tissue	Yes	Yes	qRT-PCR	CCK-8 assay, BrdU assay, Flow cytometry, luciferase reporter assay, RIP assay, Western blot.
Zhang Y et al. 2021 [86]	Plasma	Yes	Yes	qRT-PCR	Cell phenotype assays, Western blot, RNA pull-down and RIP assay, Chromatin immunoprecipitation assay.
Zhou et al. 2021 [88]	BC and normal tissue	Yes	Yes	qRT-PCR	Microarray analysis, Cell proliferation assay, Wound healing assay, Transwell assay, Western blot, Dual-luciferase reporter assay, RIP assay.
Zhu et al. 2021 [89]	BC and normal tissue	Yes	Yes	qRT-PCR	RNA interference, Cell Counting Kit-8 (CCK-8) assay, Colony formation assay, Wound healing and invasion assays, Luciferase reporter assay.
Chen et al. 2020 [91]	BC and normal tissue	Yes	Yes	qRT-PCR	Cell viability assays, Luciferase reporter assay, RIP assay, Wound-healing assay, Transwell assay, Colony formation assay, Flow cytometry, IHC, Western blot.
Ding et al. 2020 [95]	BC and normal tissue	Yes	Yes	qRT-PCR	MTT-assay, Western blot, Flow cytometry, Glycolysis analysis, Dual-luciferase reporter assay.
Fang et al. 2020 [98]	BC and normal tissue	Yes	Yes	qRT-PCR	Colony formation assay, EdU assay, Caspase-3 activity detection, Flow cytometry, Wound healing assay, Cell invasion assay, RIP assay, RNA pull-down assay, Luciferase reporter assay, IHC.
Jiang et al. 2020 [102]	BC and normal tissue	Yes	Yes	qRT-PCR	Small interfere RNA (siRNA), Cell proliferation and viability assays, Cell invasion and migration assays, Western blot, FISH, Luciferase reporter assay.
Jiang et al. 2020 [103]	BC and normal tissue	Yes	Yes	qRT-PCR	Cell proliferation assay, Transwell assay, Western blot.
Li et al. 2020 [105]	BC and normal tissue	Yes	Yes	qRT-PCR	siRNA/shRNA, Colony Formation Assay, MTSCell Viability Assay, IHC , ISH, FISH, Apoptosis, Western blot, RIP assay, RNA pull-down assay, Immunoprecipitation.
Li et al. 2020 [108]	BC and normal tissue	Yes	Yes	qRT-PCR	Cell migration, cell invasion, cell proliferation and cell apoptosis analysis, Western blot, Luciferase reporter assay.
Liu et al. 2020 [110]	BC and normal tissue	Yes	Yes	qRT-PCR	CCK-8, Flow cytometry, Luciferase reporter assay, RIP assay, Western blot.
Liu et al. 2020 [111]	BC and normal tissue	Yes	Yes	qRT-PCR	5-Ethynyl-2'-Deoxyuridine (EdU) Assay, Colony Formation Assay, Transwell assay, Flow cytometry, Luciferase Reporter Gene Assay, FISH, RIP assay, RNA pull-down assay, Immunoblotting.
Wang et al. 2020 [118]	BC and normal tissue	Yes	Yes	qRT-PCR	Western blot, Transwell assay, Wound healing assay, Cell cycle and apoptosis analysis, Colony formation assay, Cell proliferation assay, RIP assay, Luciferase activity assay, FISH.
Xiong et al. 2020 [119]	BC and normal tissue	Yes	Yes	qRT-PCR	Western blot, Luciferase reporter assay, Cell proliferation assay, Cell cycle analysis, Cell migration assay, IHC
Zhang et al. 2020 [122]	BC and normal tissue	Yes	Yes	qRT-PCR	RNA and Chromatin immunoprecipitation (RIP & CHIP), Western blot, ELISA.

Supplementary Table S2 (continued – Part 3)

Study	Type of sample	Cell lines	Animal experiments	Primary method	Other methods used
Zhao et al. 2020 [123]	BC and normal tissue	Yes	Yes	qRT-PCR	Immunohistochemistry (IHC), RNA immunoprecipitation (RIP), Western blot.
Zheng et al. 2020 [124]	BC and normal tissue	Yes	Yes	qRT-PCR	Fluorescence In Situ Hybridization (FISH), Immunohistochemistry (IHC), Western blot, RNA and Chromatin immunoprecipitation (RIP & CHIP).
Dong et al. 2019 [130]	BC and normal tissue	Yes	Yes	qRT-PCR	RNA and Chromatin immunoprecipitation (RIP & CHIP), Western blot.
Guo et al. 2019 [132]	BC and normal tissue	Yes	Yes	qRT-PCR	Fluorescence In Situ Hybridization (FISH), Western blotting, Flow cytometry.
Jiang et al. 2019 [135]	BC and normal tissue	Yes	Yes	qRT-PCR	Luciferase assay, Flow cytometry.
Li et al. 2019 [137]	BC and normal tissue	Yes	Yes	qRT-PCR	Western blotting, RNA Immunoprecipitation (RIP).
Li et al. 2019 [138]	BC and normal tissue	Yes	Yes	Microarray analysis	Reverse transcription polymerase chain reaction (RT-PCR), Luciferase assay, Western blotting, Immunohistochemistry (IHC), Flow cytometry.
Li et al. 2019 [139]	BC and normal tissue	Yes	Yes	qRT-PCR	Immunohistochemistry (IHC), Western blot.
Lv et al. 2019 [141]	BC and normal tissue	Yes	Yes	qRT-PCR	Western blotting, Luciferase assay.
Ma et al. 2019 [142]	BC and normal tissue	Yes	Yes	qRT-PCR	Immunofluoresence, FISH, IHC, Western blot, RNA and Chromatin immunoprecipitation (RIP & CHIP), Luciferase assay.
Shin et al. 2019 [147]	Blood	Yes	Yes	qRT-PCR	Western blotting, Immunohistochemistry (IHC).
Si et al. 2019 [148]	BC and normal tissue	Yes	Yes	qRT-PCR	Western blotting, Flow cytometry, Luciferase assay.
Xu et al. 2019 [156]	BC and normal tissue	Yes	Yes	qRT-PCR	Cell viability and apoptosis assay, Colony formation assay
Zhang et al. 2019 [159]	BC and normal tissue	Yes	Yes	qRT-PCR	Western blotting, Immunofluoresence.
Zheng et al. 2019 [161]	BC and normal tissue	Yes	Yes	qRT-PCR	Western blotting, Immunohistochemistry (IHC), In situ Hybridization (ISH), Immunofluorescence, Tissue microarrays (TMA).
Zheng et al. 2019 [162]	BC and normal tissue	Yes	Yes	qRT-PCR	In situ Hybridization (ISH), Western blotting, Immunohistochemistry (IHC), Flow cytometry, RNA Immunoprecipitation (RIP).
Zhou et al. 2019 [163]	BC and normal tissue	Yes	Yes	qRT-PCR	Western blotting, Luciferase assay, RNA Immunoprecipitation (RIP).

Supplementary Table S2 (continued – Part 4)

Study	Type of sample	Cell lines	Animal experiments	Primary method	Other methods used
Zhou et al. 2019 [164]	BC and normal tissue	Yes	Yes	qRT-PCR	Western blotting.
Gao et al. 2018 [167]	BC and normal tissue	Yes	Yes	qRT-PCR	Western blotting, Luciferase assay.
Li et al. 2018 [172]	BC and normal tissue	Yes	Yes	qRT-PCR	Western blotting.
Li et al. 2018 [173]	BC and normal tissue	Yes	Yes	qRT-PCR	Immunohistochemistry (IHC), Flow cytometry.
Liang et al. 2018 [174]	BC and normal tissue	Yes	Yes	qRT-PCR	Tissue Microarrays (TMA), Luciferase assay, RNA Immunoprecipitation (RIP), Immunohistochemistry (IHC).
Song et al. 2018 [178]	BC and normal tissue	Yes	Yes	qRT-PCR	Western blotting, Flow cytometry.
Wang et al. 2018 [181]	BC and normal tissue	Yes	Yes	qRT-PCR	Western blotting.
Wang et al. 2018 [182]	BC and normal tissue	Yes	Yes	qRT-PCR	Western blotting, Flow cytometry, Luciferase assay.
Yang et al. 2018 [184]	BC and normal tissue	Yes	Yes	qRT-PCR	Tissue Microarrays (TMA), Chromatin Immunoprecipitation (CHIP), Immunohistochemistry (IHC), Fluorescence In Situ Hybridization (FISH), Luciferase assay, Western blotting.
Zeng et al. 2018 [187]	BC and normal tissue	Yes	Yes	qRT-PCR	Western blotting, Luciferase assay, Flow cytometry, Immunohistochemistry (IHC).
Zhang et al. 2018 [188]	BC and normal tissue	Yes	Yes	qRT-PCR	Flow cytometry, Western blotting, Luciferase assay.
Fang et al. 2017 [194]	BC and normal tissue	Yes	Yes	qRT-PCR	Immunohistochemistry, Flow cytometry, Luciferase assay, Western blotting.
Li, et al. 2017 [196]	BC and normal tissue	Yes	Yes	qRT-PCR	Immunohistochemistry, Western blotting, Immunofluorescence, Flow cytometry.
Li et al. 2017 [197]	BC and normal tissue	Yes	Yes	qRT-PCR	Western blotting.
Sha et al. 2017 [206]	BC and normal tissue	Yes	Yes	qRT-PCR	Immunohistochemistry, Western blotting, Immunofluorescence.
Vennin et al. 2017 [208]	BC and normal tissue	Yes	Yes	qRT-PCR	Chromatin Immunoprecipitation (CHIP).

Supplementary Table S2 (continued – Part 5)

Study	Type of sample	Cell lines	Animal experiments	Primary method	Other methods used
Xu et al. 2017 [211]	BC and normal tissue	Yes	Yes	qRT-PCR	RNA Immunoprecipitation (RIP), Luciferase assay.
Zhang et al. 2017 [213]	BC and normal tissue	Yes	Yes	qRT-PCR	Luciferase assay, In situ Hybridization (ISH).
Li et al. 2016 [218]	BC and normal tissue	Yes	Yes	qRT-PCR	luciferase reporter gene assay, MTT assay, Boyden assay, flow cytometry.
Liu et al. 2016 [219]	BC and normal tissue	Yes	Yes	qRT-PCR	Immunohistochemistry, Western blotting.
Liu et al. 2015 [232]	BC and normal tissue	Yes	Yes	qRT-PCR	Flow cytometry, Western blotting, Luciferase assay.
Shi et al. 2014 [238]	BC and normal tissue	Yes	Yes	qRT-PCR	Flow cytometry, Western blotting.
She et al. 2022 [48]	BC and normal tissue	Yes	Yes	qRT-PCR	MTT assay, Transwell assay, Western blot, Immunofluorescent staining, TUNEL assay, RNA immunoprecipitation, RNA pull-down, Luciferase reporter assay, CHIP assay.
Zhou et al. 2021 [87]	Serum Extracellular Vesicles (EVs)	Yes	Yes	qRT-PCR	Western blot, MTT assay, colony formation assay, Flow cytometry, Transwell assay, Scratch test, Dual luciferase reporter assay, RNA-pull down assay.
Song et al. 2025 [176]	BC and normal tissue	Yes	Yes	qRT-PCR	Ribonuclease R (RNase R) treatment and actinomycin D assay, FISH, MTT assay, Colony formation assay, Wound healing test, Transwell assay, Dual-luciferase reporter assay, RNA immunoprecipitation (RIP), Western blot, IHC
Huang et al. 2025 [244]	BC and normal tissue	Yes	Yes	qRT-PCR	FISH, Luciferase reporter assay, Cell proliferation assay, Wound healing test, Migration and invasion, IHC, RNA immunoprecipitation (RIP) and RNA pulldown assay
Bhandari et al. 2024 [23]	BC and normal tissue	Yes	No	qRT-PCR	Cell Proliferation Assay, Western blot.
Liang et al. 2024 [26]	BC and normal tissue	Yes	No	qRT-PCR	Dual luciferase assay, Radioimmunoprecipitation assay, RNA-pull down assay, CCK-8 assay, Flow cytometry, Transwell assay.
Yi et al. 2024 [29]	BC and normal tissue	Yes	No	qRT-PCR	Flow cytometry, Transwell assay, Dual-luciferase reporter assay, Western blot, RNA immunoprecipitation assay.
Zhuang et al. 2024 [30]	BC tissue	Yes	No	RNAscope	qRT-PCR in cell lines, cell proliferation assay, transwell migration and invasion assays, IHC.
Hajibabaei et al. 2023 [33]	BC and normal tissue	Yes	No	qRT-PCR	Luciferase reporter assay, MTT assay, Cell cycle and apoptosis analysis.
Sun et al. 2023 [38]	BC tissue	Yes	No	qRT-PCR	Cell Counting Kit-8 (CCK-8) assay, Bromodeoxyuridine (BrdU) assay, RNA FISH, Western blot, Luciferase reporter assay.

Supplementary Table S2 (continued – Part 6)

Zhang et al. 2023 [41]	BC tissue	Yes	No	qRT-PCR	Western blot, in vitro cell viability assay, Cell invasion and migration assays, Cell apoptosis assay, BrdU staining, Immunofluorescence staining.
Li et al. 2022 [43]	BC and normal tissue	Yes	No	qRT-PCR	Cell cycle analysis.
Li et al. 2022 [44]	BC and normal tissue	Yes	No	qRT-PCR	Dual-luciferase reporter assay, RIP assay, Transwell assay, Western blot.
Tang et al. 2022 [50]	BC and normal tissue	Yes	No	qRT-PCR	Cell proliferation assay, Wound healing assay, Transwell assay, Colony formation assay, Cell cycle analysis, Flow cytometry, Western blot, Dual-luciferase reporter assay.
Wang et al. 2022 [51]	BC and normal tissue	Yes	No	qRT-PCR	BrdU incorporation assay (proliferation assay).
Bai et al. 2021 [55]	BC tissue	Yes	No	qRT-PCR	Cell Counting Kit-8 (CCK-8) assay, Luciferase reporter assay, Western blot, colony formation assay, cell apoptosis assay.
Chen et al. 2021 [58]	BC tissue	Yes	No	qRT-PCR	Plasmid and small interfering RNA (siRNA), CCK-8 and cell colony formation.
Dan et al. 2021 [61]	BC and normal tissue	Yes	No	qRT-PCR	Short Hairpin RNA (shRNA) Synthesis and Transfection, Cell Proliferation Assay, Colony Forming Assay, Western-Blot, Flow Cytometry, RNA FISH, Chromatin Immunoprecipitation (ChIP)-qPCR.
Huang et al. 2021 [67]	BC and normal tissue	Yes	No	qRT-PCR	RNA-binding protein immunoprecipitation (RIP) assay, western blot, Dual luciferase reporter assay, Cell apoptosis assay.
Jiang et al. 2021 [68]	BC and normal tissue	Yes	No	qRT-PCR	MTT assay and colony formation assay, Transwell assay.
Li et al. 2021 [69]	BC and normal tissue	Yes	No	qRT-PCR	Stem cell isolation, Western blot, Side population (SP) assay, Sphere formation assay, Clone formation assay, Cell counting kit-8 (CCK-8) assay, Wound healing assay, Transwell assay, Flow cytometry.
Liu et al. 2021 [70]	BC and normal tissue	Yes	No	qRT-PCR	Cell counting kit-8 (CCK-8) assay, BrdU assay, Transwell assay, TUNEL assay, RIP assay,luciferase reporter assay, Western blot.
Marcu et al. 2021 [71]	BC tissue	Yes	No	qRT-PCR	Laser capture microdissection.
Samir et al. 2021 [76]	BC and normal tissue	Yes	No	qRT-PCR	Cell Culture.
Sun et al. 2021 [79]	BC and normal tissue	Yes	No	qRT-PCR	Cell Counting Kit-8 (CCK-8) assay, Colony formation assay, Transwell assay, Wound healing assay, Western blot, Luciferase reporter assay.
Wang et al. 2021 [82]	BC and normal tissue	Yes	No	qRT-PCR	Migration and invasion assays, luciferase reporter assay, Western blot, IHC.
Xu et al. 2021 [84]	Tissue & blood BC & normal	Yes	No	lncRNA array	qRT-PCR, 5' and 3' rapid amplification of cDNA ends (RACE) and northern blot analysis, Colony formation assay, Flow cytometry analyses of apoptosis and the cell cycle.

Supplementary Table S2 (continued – Part 7)

Study	Type of sample	Cell lines	Animal experiments	Primary method	Other methods used
Xue et al. 2021 [85]	BC	Yes	No	qRT-PCR	Cell Culture.
Cheng L et al. 2020 [92]	BC and normal tissue	Yes	No	qRT-PCR	MTT assay, Colony formation assay, Wound healing assay, Transwell assay, Luciferase reporter assay, RNA pull-down assay, Western blot.
Cui et al. 2020 [93]	BC and normal tissue	Yes	No	qRT-PCR	Cell culture, Colony Formation Assay.
De Palma et al. 2020 [94]	BC and normal tissue	Yes	No	qRT-PCR	Laser-capture microdissection, High-throughput RNA sequencing.
Du et al. 2020 [96]	BC and normal tissue	Yes	No	qRT-PCR	Cell Proliferation Assay, Wound-Healing Assay, Cell Invasion Determination, Cell Apoptosis and Cell Cycle Assays, Dual-Luciferase Reporter Gene Assay, RIP assay, Western blot.
Elias-Riszk et al. 2020 [97]	benign, BC and normal tissue	Yes	No	qRT-PCR	Cell culture, RT-PCR.
Gao et al. 2020 [99]	BC and normal tissue	Yes	No	qRT-PCR	CCK-8 assay, Colony formation assay, Transwell Migration and Invasion Assays.
Gavgani et al. 2020 [100]	BC and normal tissue	Yes	No	qRT-PCR	Small interfering RNA (siRNA) transfection, cell cycle analysis, wound healing assay.
Li et al. 2020 [104]	BC and normal tissue	Yes	No	qRT-PCR	Western blot, Cell-Proliferation Assay, Transwell assay, Luciferase Reporter Assay, RIP assay, RNA pull-down assay.
Li et al. 2020 [106]	BC and normal tissue	Yes	No	qRT-PCR	Western blot, CCK-8 assay, Transwell cell invasion assay.
Li et al. 2020 [107]	BC and normal tissue	Yes	No	qRT-PCR	Colony formation assay, CCK-8 Assay, Cell apoptosis, Transwell assay, JC-1 assay, RIP assay, FISH, Immunofluorescence, Western blot, Luciferase reporter assay, RNA pull-down assay.
Lian et al. 2020 [109]	BC and normal tissue	Yes	No	qRT-PCR	Cell Proliferation Analysis, Transwell assay, RIP assay, Chromatin immunoprecipitation (ChIP) assay.
Mehrpour Layeghi et al. 2020 [112]	BC and normal tissue	Yes	No	qRT-PCR	Cell culture.
Wang et al. 2020 [114]	BC and normal tissue	Yes	No	qRT-PCR	Cell invasion assay.
Wang et al. 2020 [115]	BC and normal tissue	Yes	No	qRT-PCR	Cell Counting Kit 8 (CCK-8) Assay, Flow cytometry, Transwell assay, Luciferase reporter assay, Western blot.
Wang et al. 2020 [116]	BC and normal tissue	Yes	No	RT-PCR	Flow cytometry, Luciferase assay.
Wang et al. 2020 [117]	BC and normal tissue	Yes	No	qRT-PCR	Western blot, RIP assay, RNA-RNA in vitro interaction assay, mRNA stability analysis, Sphere-formation analysis, Cell viability assay.

Supplementary Table S2 (continued – Part 8)

Study	Type of sample	Cell lines	Animal experiments	Primary method	Other methods used
Yu et al. 2020 [121]	BC and normal tissue	Yes	No	qRT-PCR	Cell Counting Kit-8 (CCK-8), Apoptosis detection by flow cytometry.
Beltrán-Anaya et al. 2019 [125]	BC tissue	Yes	No	qRT-PCR	Tissue Microarrays (TMA).
Calanca et al. 2019 [126]	BC tissue	Yes	No	qRT-PCR	Methylation specific-polymerase chain reaction (MS-PCR), Immunohistochemistry (IHC).
Cao et al. 2019 [127]	BC and normal tissue	Yes	No	qRT-PCR	Western blotting.
Chen et al. 2019 [128]	BC and normal tissue	Yes	No	qRT-PCR	Western blotting, Immunofluoresence.
Ding et al. 2019 [129]	BC and normal tissue	Yes	No	qRT-PCR	Luciferase assay, Western blotting.
Gao et al. 2019 [131]	BC and normal tissue	Yes	No	qRT-PCR	Luciferase assay.
Guo, Lv et al. 2019 [133]	BC and normal tissue	Yes	No	qRT-PCR	Western blotting.
Hu et al. 2019 [134]	BC and normal tissue	Yes	No	qRT-PCR	Western blotting, Luciferase assay.
Keshavarz and Asadi 2019 [136]	BC and normal tissue	Yes	No	qRT-PCR	Western blotting.
Liu et al. 2019 [140]	BC and normal tissue	Yes	No	qRT-PCR	Luciferase assay.
Mehta-Mujoo et al. 2019 [143]	BC and normal tissue	Yes	No	qRT-PCR	In situ Hybridization (ISH).
Pang et al. 2019 [144]	BC and normal tissue	Yes	No	qRT-PCR	Western blotting.
Ravanbakhsh Gavgani et al. 2019 [145]	BC and normal tissue	Yes	No	qRT-PCR	Sanger sequencing.
Song et al. 2019 [149]	BC and normal tissue	Yes	No	qRT-PCR	Cell culture, Western blotting.
Sun et al. 2019 [150]	BC and normal tissue	Yes	No	qRT-PCR	Cell culture, cell viability.
Wang, Liu et al. 2019 [151]	Plasma	Yes	No	qRT-PCR	Western blotting, ELISA.

Supplementary Table S2 (continued – Part 9)

Study	Type of sample	Cell lines	Animal experiments	Primary method	Other methods used
Wang et al. 2019 [152]	BC and normal tissue	Yes	No	qRT-PCR	Cell culture, Transwell assay.
Wei et al. 2019 [153]	Tissue & Plasma BC & normal	Yes	No	qRT-PCR	Western blotting.
Xie et al. 2019 [155]	BC and normal tissue	Yes	No	qRT-PCR	Luciferase assay, Western blotting.
Yang et al. 2019 [157]	BC and normal tissue	Yes	No	qRT-PCR	Luciferase assay, Flow cytometry.
Yu et al. 2019 [158]	BC and normal tissue	Yes	No	qRT-PCR	Cell culture, cell proliferation assay.
Zhao et al. 2019 [160]	BC and normal tissue	Yes	No	qRT-PCR	Luciferase assay, Western blotting.
Zhu et al. 2019 [165]	BC and normal tissue	Yes	No	qRT-PCR	Fluorescence In Situ Hybridization (FISH), Western blotting.
Dong et al. 2018 [166]	BC and normal tissue	Yes	No	qRT-PCR	Chromatic Immunoprecipitation (CHIP), Immunofluorescence, Fluorescence In Situ Hybridization (FISH), Immunohistochemistry (IHC), Western blotting.
Jeong et al. 2018 [168]	BC and normal tissue	Yes	No	qRT-PCR	Flow cytometry, Genome wide expression analysis.
Jiang et al. 2018 [169]	BC and normal tissue	Yes	No	qRT-PCR	Cell proliferation assays, Cell migration and invasion assays.
Li et al. 2018 [170]	BC tissue	Yes	No	qRT-PCR	Western blotting.
Li et al. 2018 [171]	BC and normal tissue	Yes	No	qRT-PCR	Western blotting, RNA Immunoprecipitation (RIP), Luciferase assay.
Lou et al. 2018 [176]	BC and normal tissue	Yes	No	qRT-PCR	Western blotting, Flow cytometry.
Tang et al. 2018 [179]	BC and normal tissue	Yes	No	qRT-PCR	Western blotting, Luciferase assay.
Tao et al. 2018 [180]	BC and normal tissue	Yes	No	qRT-PCR	Western blotting, Luciferase assay, RNA Immunoprecipitation (RIP).
Xia et al. 2018 [183]	BC and normal tissue	Yes	No	qRT-PCR	Western blotting.
Yang et al. 2018 [185]	BC and normal tissue	Yes	No	qRT-PCR	Western blotting.

Supplementary Table S2 (continued – Part 10)

Study	Type of sample	Cell lines	Animal experiments	Primary method	Other methods used
Yang et al. 2018 [186]	BC and normal tissue	Yes	No	qRT-PCR	Tissue Microarrays (TMA), Flow cytometry.
Zhang et al. 2018 [189]	BC and normal tissue	Yes	No	qRT-PCR	Western blotting, Luciferase assay, Flow cytometry.
Zheng et al. 2018 [190]	BC and normal tissue	Yes	No	qRT-PCR	Western blotting, Luciferase assay.
Chen et al. 2017 [192]	BC and normal tissue	Yes	No	qRT-PCR	Western blotting.
Fan et al. 2017 [193]	BC and normal tissue	Yes	No	qRT-PCR	Western blotting, Flow cytometry.
Li et al. 2017 [198]	BC and normal tissue	Yes	No	qRT-PCR	Western blotting, Luciferase assay.
Li et al. 2017 [199]	BC and normal tissue	Yes	No	qRT-PCR	siRNA transfection, Cell proliferation analysis, Transwell assay, Cell apoptosis analysis.
Li et al. 2017 [201]	BC tissue	Yes	No	qRT-PCR	Cell growth assay, Apoptosis assay.
Liu et al. 2017 [202]	BC and normal tissue	Yes	No	qRT-PCR	Flow cytometry, Western blotting.
Liu et al. 2017 [203]	BC and normal tissue	Yes	No	qRT-PCR	Luciferase assay.
Qian et al. 2017 [204]	BC and normal tissue	Yes	No	qRT-PCR	Western blotting, Luciferase assay.
Wu et al. 2017 [209]	BC and normal tissue	Yes	No	qRT-PCR	Western blotting.
Xi et al. 2017 [210]	BC and normal tissue	Yes	No	qRT-PCR	Western blotting, Microarrays, Chromatin Immunoprecipitation (CHIP), Mass spectrometry, Fluorescence in situ hybridization (FISH).
Zuo et al. 2017 [215]	BC and normal tissue	Yes	No	qRT-PCR	Immunohistochemistry (IHC), Luciferase assay.
Huang et al. 2016 [216]	BC and normal tissue	Yes	No	qRT-PCR	Cell culture.
Lei et al. 2016 [217]	BC and normal tissue	Yes	No	qRT-PCR	Flow cytometry.
Miao et al. 2016 [220]	BC & normal tissue, Serum	Yes	No	qRT-PCR	Flow Cytometry.

Supplementary Table S2 (continued – Part 11)

Sun et al. 2016 [221]	BC and normal tissue	Yes	No	qRT-PCR	Flow cytometry, Chromatin immunoprecipitation (ChIP), In situ hybridization (ISH).
Xu et al. 2016 [223]	BC and normal tissue	Yes	No	qRT-PCR	Flow cytometry.
Yang et al. 2016 [224]	BC and normal tissue	Yes	No	qRT-PCR	small interfering RNA (siRNA), colony formation and MTT assay, Wound healing assay, Transwell assay.
Yang et al. 2016 [226]	BC and normal tissue	Yes	No	qRT-PCR	siRNA, Immunoblotting and antibodies, Cell proliferation assay, Colony formation assay.
Chen et al. 2015 [229]	BC and normal tissue	Yes	No	qRT-PCR	LncRNA expression profiling, siRNA transfection, Cell proliferation assay, Cell Invasion assay.
He et al. 2015 [230]	BC and normal tissue	Yes	No	qRT-PCR	Western blotting.
Kang et al. 2015 [231]	BC and normal tissue	Yes	No	qRT-PCR	Gene strand-specific RT-PCR, DNA methylation analysis.
Liu et al. 2015 [233]	BC and normal tissue	Yes	No	qRT-PCR	siRNA transfection, Cell proliferation assay, Wound healing assay, MAtrigel invasion assay.
Redis et al. 2013 [241]	BC and normal tissue	Yes	No	qRT-PCR	Tissue Microarrays, In Situ Hybridization (ISH), Bionformatics.
Cooper et al. 2009 [242]	BC and normal tissue	Yes	No	qRT-PCR	RT–PCR and triple-primer-PCR, Western blot, Cell proliferation assay.
Fan et al. 2025 [243]	BC and normal tissue	Yes	No	qRT-PCR	siRNA transfection, Western blotting,Transcriptional reporter assays, F-actin staining, MTS cell proliferation assays, RNA pull-down, Transwell migration assays, RNA immunoprecipitation, Proximity ligation analysis, Embryonic zebrafish extravasation assay, Chromatin immunoprecipitation assays.

BC = Breast cancer; qRT-PCR = quantitative reverse transcription polymerase chain reaction.

Zafrakas et al. The role of long non-coding RNA in breast cancer diagnosis and treatment based on evidence from clinical studies: A scoping review.

Supplementary Table S3. PRISMA-ScR Checklist

**Section**	**Item**	**PRISMA-ScR Checklist Item**	**Yes/No**
**Title**	1	Identify the report as a scoping review.	Yes
**Abstract**			
Structured summary	2	Provide a structured summary that includes (as applicable) background, objectives, eligibility criteria, sources of evidence, charting methods, results, and conclusions that relate to the review questions and objectives.	Yes
**Introduction**			
Rationale	3	Describe the rationale for the review in the context of what is already known. Explain why the review questions/objectives lend themselves to a scoping review approach.	Yes
Objectives	4	Provide an explicit statement of the questions and objectives being addressed with reference to their key elements (e.g., population or participants, concepts, and context) or other relevant key elements used to conceptualize the review questions and/or objectives.	Yes
**Methods**			
Protocol and registration	5	Indicate whether a review protocol exists; state if and where it can be accessed (e.g., a Web address); and if available, provide registration information, including the registration number.	Yes
Eligibility criteria	6	Specify characteristics of the sources of evidence used as eligibility criteria (e.g., years considered, language, and publication status), and provide a rationale.	Yes
Information sources	7	Describe all information sources in the search (e.g., databases with dates of coverage and contact with authors to identify additional sources), as well as the date the most recent search was executed.	Yes
Search	8	Present the full electronic search strategy for at least 1 database, including any limits used, such that it could be repeated.	Yes
Selection of sources of Evidence	9	State the process for selecting sources of evidence (i.e., screening and eligibility) included in the scoping review.	Yes
Data charting process	10	Describe the methods of charting data from the included sources of evidence (e.g., calibrated forms or forms that have been tested by the team before their use, and whether data charting was done independently or in duplicate) and any processes for obtaining and confirming data from investigators.	Yes
Data items	11	List and define all variables for which data were sought and any assumptions and simplifications made.	Yes
Critical appraisal of individual sources of evidence	12	If done, provide a rationale for conducting a critical appraisal of included sources of evidence; describe the methods used and how this information was used in any data synthesis (if appropriate).	No
Summary measures	13	Not applicable for scoping reviews.	No
Synthesis of results	14	Describe the methods of handling and summarizing the data that were charted.	Yes
Risk of bias across studies	15	Not applicable for scoping reviews.	No
Additional analyses	16	Not applicable for scoping reviews.	No

Supplementary Table S3 (continued)

**Results**			
Selection of sources of evidence	17	Give numbers of sources of evidence screened, assessed for eligibility, and included in the review, with reasons for exclusions at each stage, ideally using a flow diagram.	Yes
Characteristics of sources of evidence	18	For each source of evidence, present characteristics for which data were charted and provide the citations.	Yes
Critical appraisal within sources of evidence	19	If done, present data on critical appraisal of included sources of evidence (see item 12).	No
Results of individual sources of evidence	20	For each included source of evidence, present the relevant data that were charted that relate to the review questions and objectives.	Yes
Synthesis of results	21	Summarize and/or present the charting results as they relate to the review questions and objectives.	Yes
Risk of bias across studies	22	Not applicable for scoping reviews.	No
Additional analyses	23	Not applicable for scoping reviews.	No
**Discussion**			
Summary of evidence	24	Summarize the main results (including an overview of concepts, themes, and types of evidence available), link to the review questions and objectives, , and consider the relevance to key groups.	Yes
Limitations	25	Discuss the limitations of the scoping review process.	Yes
Conclusions	26	Provide a general interpretation of the results with respect to the review questions and objectives, as well as potential implications and/or next steps.	Yes
**Funding**	27	Describe sources of funding for the included sources of evidence, as well as sources of funding for the scoping review. Describe the role of the funders of the scoping review.	Yes
